# Design and development of a novel polymer coating system with exceptional creep resistance

**DOI:** 10.1038/s44296-025-00063-x

**Published:** 2025-06-30

**Authors:** Nader Ameli, Jaya Verma, Beth Muthoni Irungu, Sepideh Aliasghari, Andrei Shishkin, Allan Matthews, Saurav Goel

**Affiliations:** 1 School of Engineering, London South Bank University, London, UK; 2 Henry Royce Institute, The University of Manchester, Manchester, UK; 3 Institute of Physics and Materials Science, Natural Sciences and Technology Department at Riga Technical University, 3 P. Valdena Str., Riga, LV-1048 Latvia; 4 Department of Mechanical Engineering, University of Petroleum and Energy Studies, Dehradun, India

**Keywords:** Engineering, Nanoscience and technology

## Abstract

Polymer coatings often suffer from poor mechanical properties, including low strength and modulus, making them prone to creep failure under minimal loads. To address these challenges, this study introduces a novel polyurethane (PU) coating reinforced with 4 wt% hollow ceramic microspheres (HCM) coated with a TiO₂ shell (HCM@TiO₂). The modified coating exhibited a 111% increase in nanoindentation hardness, along with significant reductions in creep displacement (31%), indentation creep rate (19%), and creep strain rate sensitivity (28%) compared to the base PU. In contrast, a second additive, solid silica nanospheres with TiO₂ shells (SSN@TiO₂), did not improve mechanical performance and even increased creep displacement by 31%, likely due to polymer chain sliding. Notably, the HCM@TiO₂ coating maintained and even improved its creep resistance under higher loads. These findings suggest that HCM@TiO₂-enhanced coatings could be highly beneficial for applications requiring resistance to high-cycle creep-fatigue failure.

## Introduction

Polymer coatings find extensive applications across various industries including medical, automotive, construction, maritime, and electronics – thanks to their exceptional properties that help to protect surfaces against mechanical, corrosion, optical and even biological damage. Among these, polyurethane (Pure PU) coatings have garnered significant attention due to their nonflammability, ease of manufacture, neutrality and economic price. Chungprempree et al.^1^ demonstrated the efficacy of polymer coating in the marine sector. Similarly, Shah et al.^2^ highlighted the superior applications of polymer coatings, particularly Pure PU, in the automobile industry. Furthermore, recent research has shown promising advancements in the biomedical sector, with investigations confirming the suitability of Pure PU coatings for biomedical components due to their excellent biocompatibility and biodegradability^3–5^.

However, polymeric coatings possess significant limitations. Due to their soft nature in comparison to ceramic and metal coatings, they are susceptible to issues such as delamination^6^, erosion^7^, microcracking^8^, scratches^9^ and creep failure^10,11^. Specifically, creep arises from the deformation of polymer molecular chains and the subsequent viscoelastic flow, under a constant load or stress (even at significantly lower loads than the yield stress). These attributes adversely affect the design rule due to the susceptibility of coating failure at low operating loads which limits long-term durability and reliability of polymer coatings^12^.

To overcome these limitations, several modifications have been proposed by the researchers. One approach involves the incorporation of micro- and nanoparticle additives into the polymer to enhance thermal and mechanical properties^13^. Across a wide array of additives employed, SiO_2_, TiO_2_ and ZnO have stood out as prominent choices. Notably, nano-SiO_2_ demonstrates significant enhancements in material hardness, strength, stability and resistance to creep^14,15^.

Alameri and Oltulu^16^ demonstrated that a mere 1% addition of nano-silica to polymer composite led to a substantial increase in the modulus and compressive strength. In another work by Rajabimashhadi et al.^17^, polyurethane coatings were reinforced with nano, micro and hybrid SiO_2_ particles, revealing that hybrid fillers offer superior hydrophobic and mechanical properties. Meanwhile, nano-TiO_2_, as a nanofiller in polymer exhibits a range of beneficial effects on substrates, including enhanced thermal stability, crystallinity and free volume parameters, making it a widely used polymer modifier^18,19^. Li et al.^20^ proposed the addition of nano-TiO_2_ particles into three types of polymer coatings- polyurethane, epoxy resin and chlorinated rubber and noticed a significant reduction in the micro defects. In addition to these, nano-ZnO also plays a crucial role in enhancing polymer characteristics by enhancing physical and chemical stability, optical conductivity and corrosion resistance^21–23^.

It is worth noting that while the addition of micro- and nanoparticles can enhance functional properties such as contact angle, antimicrobial protection and anti-corrosiveness, it does not necessarily imply an improvement in the mechanical behaviour, especially creep resistance. In other words, if the size, concentration, incorporation conditions and their interaction with the base material are not carefully selected, these nanoparticles can potentially have adverse effects, leading to deteriorated strength of the primary substrate^24,25^.

Among many approaches, the proposition of core-shell (CS) functional particles has gained stronger traction in the research community due to its compelling advantages. These particles consist of a core, covered by a secondary composition forming their exterior shell^26^. CS can be produced in both nano and micro dimensions based on the methods of preparation. In recent years there has been a growing interest in the use of core-shell particles in polymer coatings^27–29^. Many of these composite coatings not only enhance mechanical and chemical properties (such as increasing the strength of coatings^30^, wear resistance^31^, and erosion resistance^32^) but also simultaneously exhibit exceptional biological properties (such as antimicrobial^33^ and anti-algal effects^34^). However, while core-shell particles have the potential to leverage the advantages of both components, they may exhibit behaviour that differs significantly from their constituent elements^35–37^. For this reason, employing the appropriate combinations and thoroughly evaluating their properties for the specific application is essential.

Significant attention has been paid to prior studies that have predominantly concentrated on the biological effects of core-shell particles in polymers, specifically within polyurethane coatings. On the other hand, researchers investigating the mechanical characteristics of these particles have encountered limitations due to the thin size of the coating layer^38,39^. To compensate for this limitation, nanoindentation methods offer an attractive choice for testing. Nanoindentation (ISO 14577 and ASTM E2546–07) serves as a robust tool for assessing the mechanical properties of polymer coatings. Through this method, various characteristics of thin and soft materials, including hardness, Young’s modulus, yield stress, fracture toughness and creep behaviour can be evaluated with ease. Another advantage of this technique is its ability to achieve reliable results without requiring a large work sample, unlike traditional testing methods. However, only a few researchers have utilised this method to investigate the creep behaviour in polymer coatings. Interestingly, no prior work has been done to assess the creep resistance of Pure PU coating or additive-incorporated PU coating.

The objective of this paper was to examine the creep behaviour of newly developed coatings and to benchmark them to several other compositions by leveraging the principle of core-shell micro/nanoparticles. To this end, standard nanoindentation creep tests as per ISO 14577-1:2015 were conducted to investigate the effect of indentation load and durations on the coating performance. Through this work, we provide novel results on the creep displacement, indentation rate, strain rate sensitivity and nanomechanical properties of pure PU and additive-containing PU coatings involving materials such as SiO_2_ and TiO_2_. The findings from this study provide promising insights into the potential applications of the novel polymer coatings reported in this work.

## Results

### Characterisation of materials

**Fig. 1:**
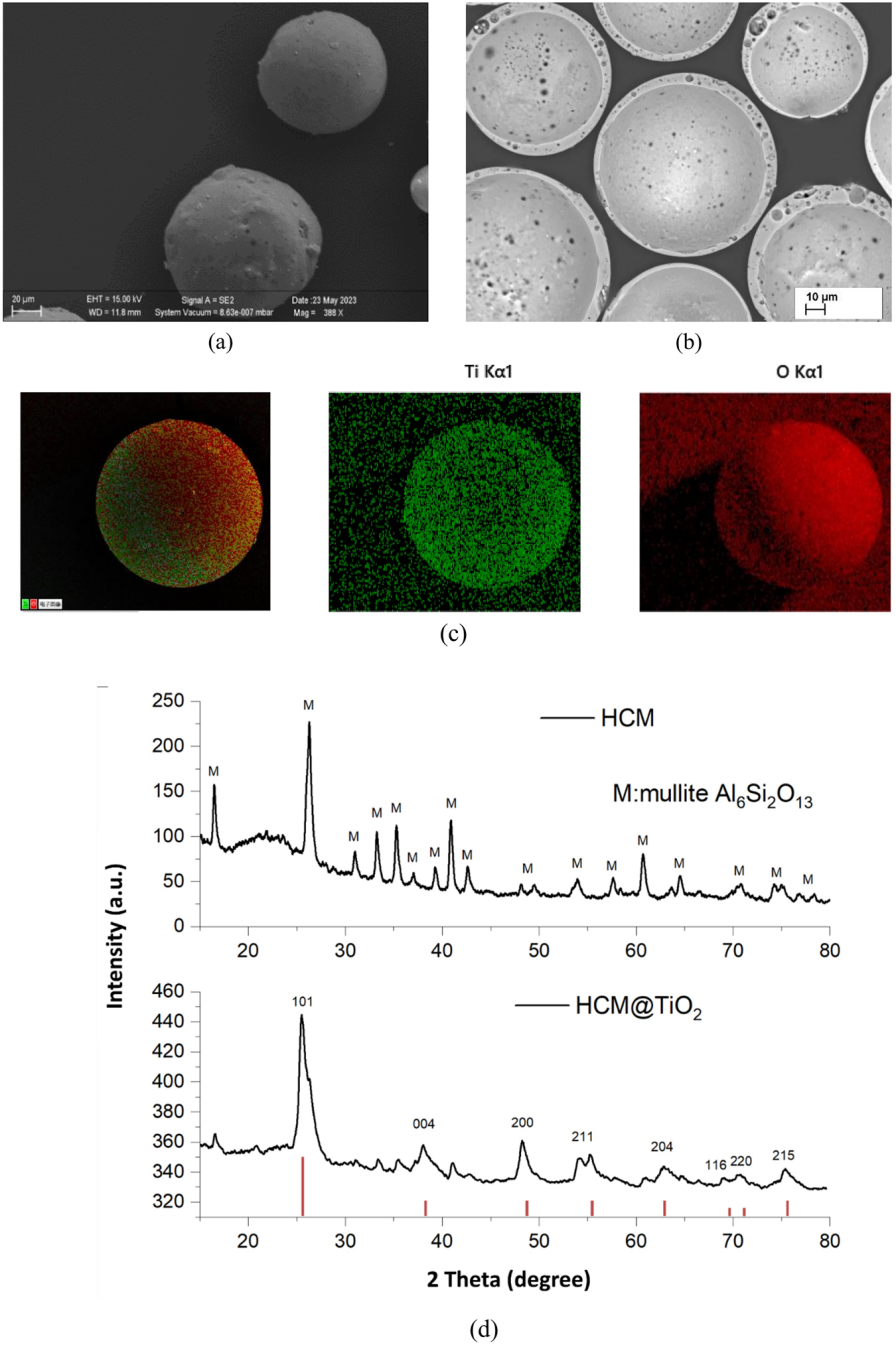
Morphology and structural characterisation of HCM and HCM@TiO₂ core-shell particles. **a** SEM image of hollow ceramic microspheres (HCMs), **b** SEM cross-section of the HCM@TiO_2_ core-shell particles, **c** EDS analysis showing elemental composition of the core-shell particles (red markings as per JCPDS 21-1272 revealed and confirmed anatase phase of titania), **d** XRD spectra of HCM and HCM@TiO_2_ core-shell particles.

In the SEM images (Fig. 1a, b), the HCM particles exhibit distinct globular characteristics on their surface, with much of the surface showing embossing and perforations. The SEM images further revealed that most of the core-shell microspheres were spherical, with the hollow ceramic particles measuring approximately 65 µm and the core-shell microparticles measuring around 70 µm. Figure 1c shows the EDS results indicating the elements present on the core-shell particles. Figure 1d shows the XRD spectra of the HCM and HCM@TiO_2_ particles. The prominent peaks shown in Fig. 1d were resolved to gain information about the phase and to demonstrate the TiO_2_ phase information. The XRD patterns confirm the uniform deposition of the TiO_2_ shell on the HCM particles. The XRD analysis identified the composition of HCM as mullite (Al_6_Si_2_O_13_) and the shell deposited on the HCM core as anatase-phase titania (TiO_2_). This was further validated by the JCPDS 21-1272 reference markings displayed at the bottom of the spectra.

**Fig. 2:**
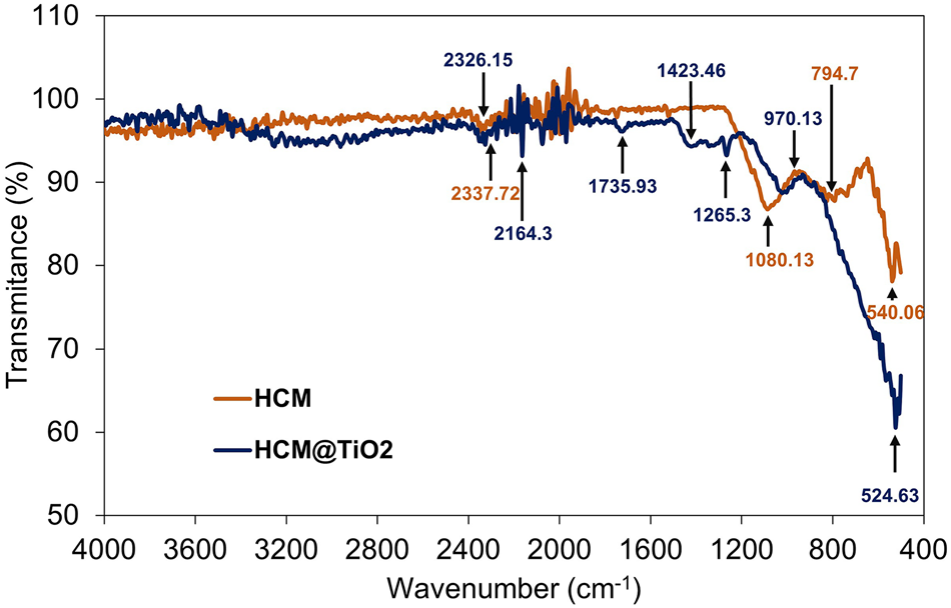
FTIR spectra of HCM and HCM@TiO_2_ CS particles.

The FTIR spectra of both HCM and HCM@TiO_2_ core-shell particles are shown in Fig. 2. Notably, three distinct peaks emerged at 1080.13, 794.7 and 540.06 cm^−^^1^, corresponding to significant vibrations and Si-O-Si bending bonds, indicative of silica network formation via the concentration of silanols to Si-O-Si. Furthermore, in the spectra of core-shell particles, a sharp peak at 970.13 cm^−1^ was observed, corresponding to the Si-O-Ti bond, suggesting the successful formation of a TiO_2_ layer on the hollow ceramic core, i.e., HCM@TiO_2_ core-shell structure. Conversely, bands at 2337.72 cm^−1^ were detected, attributable to the stretching and bending of H-O-H due to absorbed water. The O-H stretching vibration of hydroxyl groups was represented by the band at 2326.15 cm^−1^, while peaks at 1423.46, 1735.93, and 1265.3 cm^−1^ depicted water Ti-OH and Ti-O modes.

### Creep displacement

**Fig. 3:**
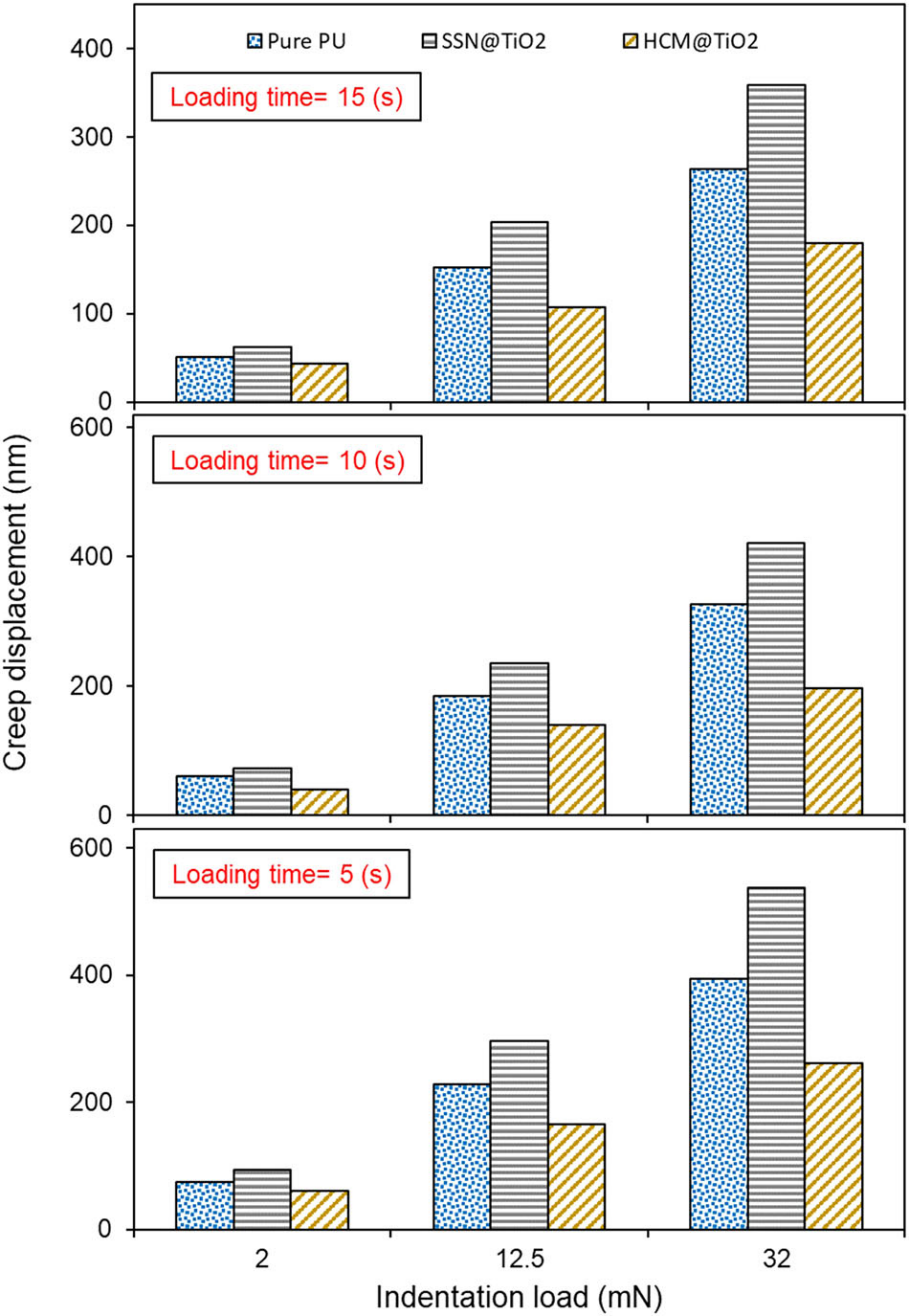
Effect of indentation load over various loading times on creep displacement.

Figure 3 shows the trends of creep displacement over different indentation loads for each coating. These values indicate the displacement of the indenter during holding time. Moreover, the charts for different loading times are provided as supplementary information (see Figs. S1 to S3). It may be seen from Fig. 3 that creep displacement increases with increasing indentation load. Besides, increasing loading time leads to a reduction in creep displacement. However, it should be noted that the general behaviour of each coating is almost identical under different loading times. These general trends are consistent with previously reported results in literature^40,41^.

Of interest is that the HCM@TiO_2_ coating consistently showed the largest creep resistance. More importantly, while other coatings showed quasi-linear rise in the indentation load, HCM@TiO_2_ coating demonstrated a higher level of resistance. For instance, the increase in creep displacement for HCM@TiO_2_ between the indentation load of 12.5 mN to 32 mN was 34%, which was less than the Pure PU coating with 75% augmentation.

Conversely, the SSN@TiO_2_ coating consistently demonstrated a high degree of creep compared to other coatings, suggesting that the SSN core did not work in favour of creep performance of the pure PU. On average, the creep displacement for this coating exceeded that of Pure PU by approximately 31%.

### Indentation creep rate (C_IT_%)

To quantify creep rate for a more effective comparison, the concept of indentation creep rate was used^42^ which can be expressed as:

1 $${C}_{{IT}} \% =\frac{{h}_{\max }-{h}_{l}}{{h}_{l}}\times 100$$

where *h*_*l*_ is the maximum displacement during the loading stage and *h*_*max*_ is the indentation depth at the end of holding stage. A bigger *C*_*IT*_ value denotes a higher creep deformation.

**Fig. 4:**
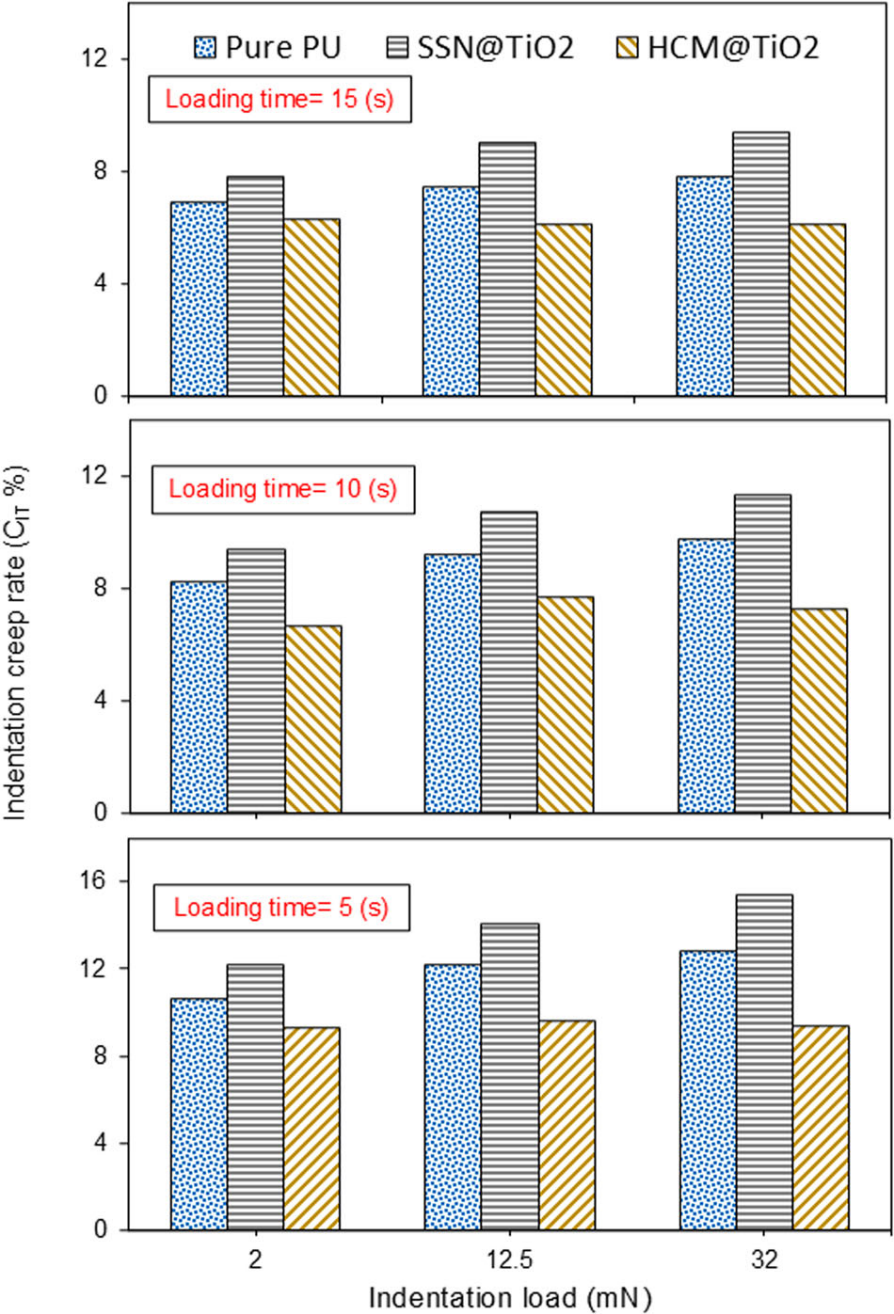
Indentation creep rate (*C*_*IT*_ *%*) versus indentation load for all four samples for various loading durations.

Figure 4 demonstrates the changes in indentation creep rate at different loads and loading times. The individual charts (for each indentation load and loading time) can be found in the supplementary as Figs. S4–S6.

As a general trend, *C*_*IT*_ slightly rises with the increasing indentation load and declines with the increasing loading duration. Furthermore, SSN@TiO_2_ coatings showed the largest indentation creep rate, which is consistent with the observations made about the creep deformation in the previous section. On average, the *C*_*IT*_ shown by SSN@TiO_2_ was 17% higher than Pure PU.

However, HCM@TiO_2_ coating revealed the lowest indentation creep rate for all loads at all durations. Its creep resistance was 21% higher than the pure PU coating. More importantly, HCM@TiO_2_ showed higher creep strength at higher indentation loads. That is, the indentation creep rate of HCM@TiO_2_ coating decreases as the indentation load increases, contrary to what might be expected. This counter-institutive behaviour contrasts with that of most polymer coatings investigated in previous investigations. We proposed, based on this evidence, that the polymer coating system we developed in this work can be used for applications requiring high-cycle fatigue loading.

### Creep strain rate sensitivity (m)

Among the different criteria for investigation of the creep behaviour of polymer coatings, creep strain rate sensitivity (*m*) is a well-known parameter that provides beneficial information regarding the creep deformation mechanism^43,44^. It is defined as^45^:

2 $$m=\frac{d\left(\log \sigma \right)}{d\left(\log \dot{\varepsilon }\right)}$$

where *σ* and $\dot{\varepsilon }$ are stress and creep strain rate, respectively. Typically, the higher *m* value shows that the material is more susceptible to deformation over time when subjected to a load and may exhibit a stronger reliance on specific loading conditions. Hence, the lower *m* would represent the higher creep resistance^46^.

The stress in the material could be calculated as:

3 $$\sigma =\frac{{P}_{\max }}{{A}_{p}\left({h}_{c}\right)}$$

where *P*_*max*_ refers to the maximum load applied during indentation, while ${A}_{p}\left({h}_{c}\right)$ represents the residual projected area of contact left by the indenter^47^. ${A}_{p}\left({h}_{c}\right)$ is a function of the distance *h*_*c*_ from the tip, and their relationship could be simply derived based on the indenter geometry. For an ideal Berkovich indenter, it can be calculated as ${A}_{p}\left({h}_{c}\right)=24.5{{h}_{c}}^{2}$. According to the instrument manufacturer, the indenter was categorised as *very sharp*, and the following equation has been suggested for the sharp Berkovich indenter^48^.

4 $${A}_{p}\left({h}_{c}\right)=24.5{{h}_{c}}^{2}+800{h}_{c}$$

The unit of ${h}_{c}$ in this equation must be in nanometre (which gives us the unit of ${A}_{p}\left({h}_{c}\right)$ as nm^2^). Otherwise, provided that the desire is to use the metre unit, the coefficient of ${h}_{c}$ should be replaced with 800×10^-18^.

Next, the creep strain rate can be calculated as^49^:

5 $$\dot{\varepsilon }=\frac{1}{h}\frac{{dh}}{{dt}}$$

where *h* and *t* are the instantaneous indentation depth and indentation time, respectively.

Thus, the equation for creep strain rate sensitivity can be expressed as follows:

6 $$m=\frac{d\left(\log \left(\frac{{P}_{\max }}{24.5{{h}_{c}}^{2}+800{h}_{c}}\right)\right)}{d\left(\log \frac{1}{h}\frac{{dh}}{{dt}}\right)}$$

Log (*σ*) versus Log ($\dot{\varepsilon })$ plots for 27 distinct conditions of this research are provided in Figs. S7–S15. The different *m* values extracted from these plots are summarised in Fig. 5.

**Fig. 5:**
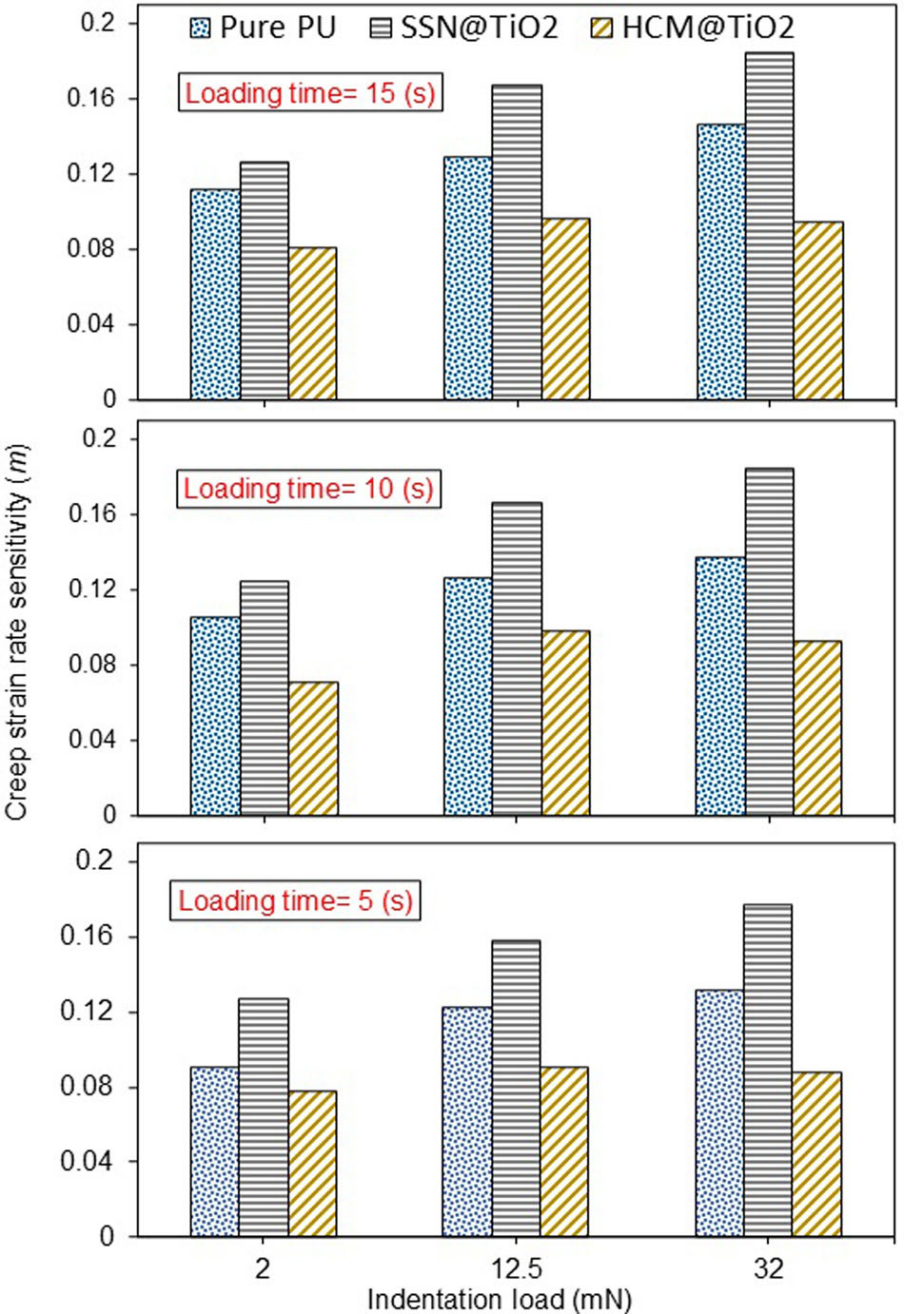
Effect of indentation load over various loading times on creep strain rate sensitivity.

The most notable observation from these plots is that the creep strain rate sensitivity was consistently minimum for the HCM@TiO_2_ coating. On average, the *m* value for the HCM@TiO_2_ coating was 28% and 44% lower than the Pure PU and SSN@TiO_2_, respectively. Additionally, in general, the *m* values were observed to increase with the rising indentation load. An intriguing observation, however, was that the behaviour of the HCM@TiO_2_ coating did not follow the same trend. While the increase in *m* value from indentation force of 12.5 mN to 32 mN for Pure PU and SSN@TiO_2_ coatings was 9.7% and 11.3%, respectively, the HCM@TiO_2_ coating experienced 3.4% reduction in the *m* value. The significance of this matter is accentuated when there is a concurrent requirement for resistance against both creep and fatigue. Sectors such as bioengineering^50^, marine^51^, and power generation^52^ stand out as crucial domains necessitating this dual functionality.

The SSN@TiO_2_ coating exhibits the lowest resistance to creep compared to the other coatings. Furthermore, it showed the most pronounced increase in the *m* value in contrast to its counterparts. This finding demonstrates that although SSN@TiO_2_ coating could present valuable improvements in antifungal effect^53^, anti-scratch behaviour^54^, and compressive strength^55^, it might not be a very good candidate for applications undergoing fatigue-creep loading.

Fig. S16 (Supplementary information) can be referred to observe the impact of loading time on creep strain rate sensitivity. There was only a very weak tendency for the increase *m* value by increasing loading time. Therefore, increasing the loading time from 5 s to 15 s results in a mere 12.4%, 3.7%, and 5.8% rise in creep strain rate sensitivity for Pure PU, SSN@TiO_2_, and HCM@TiO_2_ coatings, respectively. Thus, it can be inferred that loading time does not significantly impact the *m* value (Eq. (6)).

### Nanomechanical properties

**Fig. 6:**
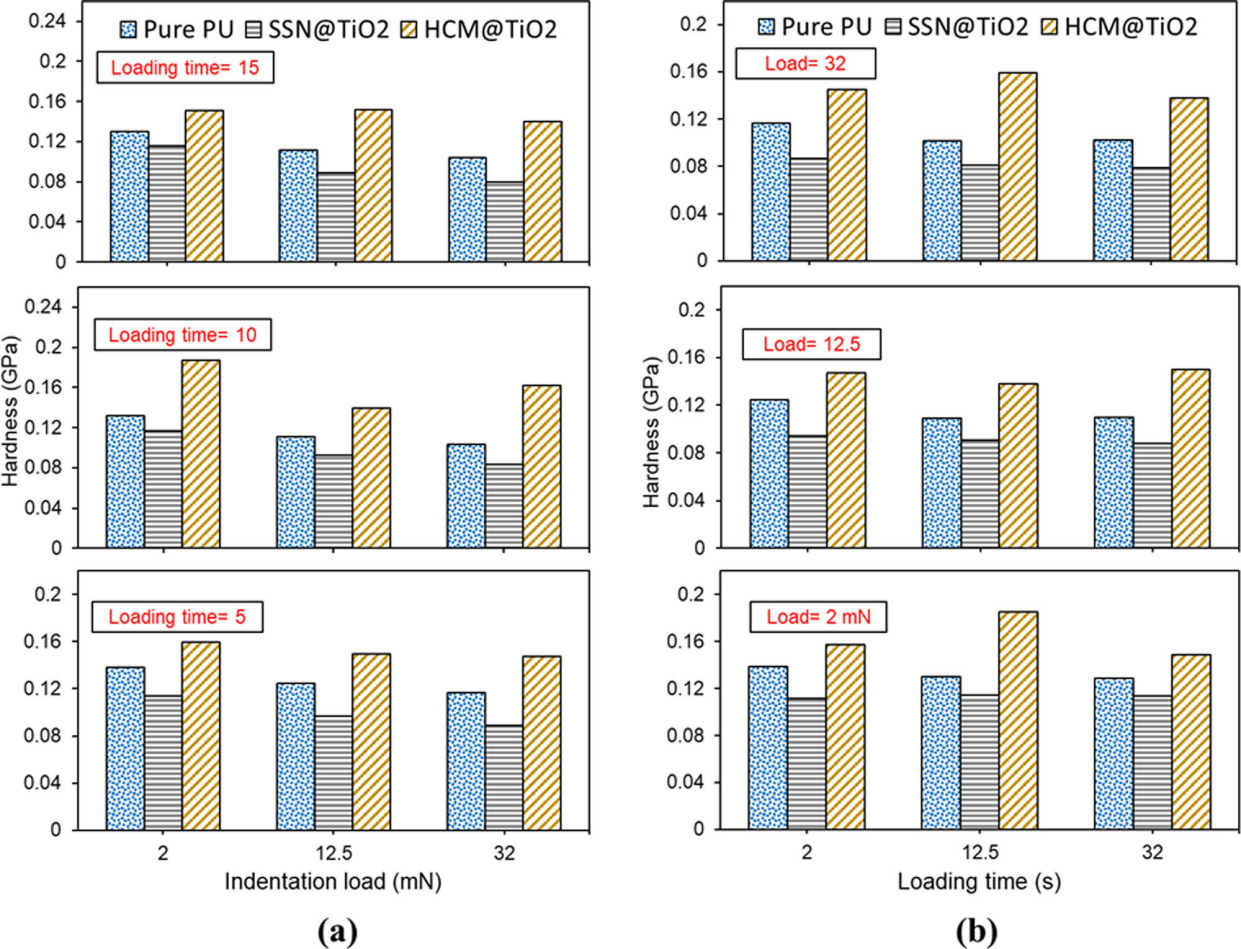
Variation of hardness under different conditions **a** indentation loads, and **b** indentation time.

Figure 6 illustrates how indentation load and duration affect the instantaneous hardness estimated using Eqs. (3) and (4) by considering only the dwell period load-displacement data for different coatings. Here, the HCM@TiO_2_ coating revealed the highest hardness value, while the SSN@TiO_2_ coating demonstrated the lowest. It suggests that the low creep displacement (high creep resistance) for HCM@TiO_2_ indicated its high hardness. The literature also supports the inverse relationship between hardness and creep displacement^56^, indentation creep rate^57^, and creep strain rate sensitivity^46^. Despite the general trend of decreasing hardness with increasing indentation load, the HCM@TiO_2_ coating showed a contrary behaviour at higher loads. While increasing the indentation load from 12.5 mN to 32 mN a reduction of hardness for the Pure PU and SSN@TiO_2_ coatings by 10% and 14%, respectively was observed compared to 13.8% increasing hardness of the HCM@TiO_2_ coating.

The other distinct behaviour observed for the HCM@TiO_2_ coating was with the increase in the loading time (Fig. 6b). While the variation in indentation time has minimal effect on the hardness of other coatings, the hardness of the HCM@TiO_2_ coating increased by an average of 5%.

**Fig. 7:**
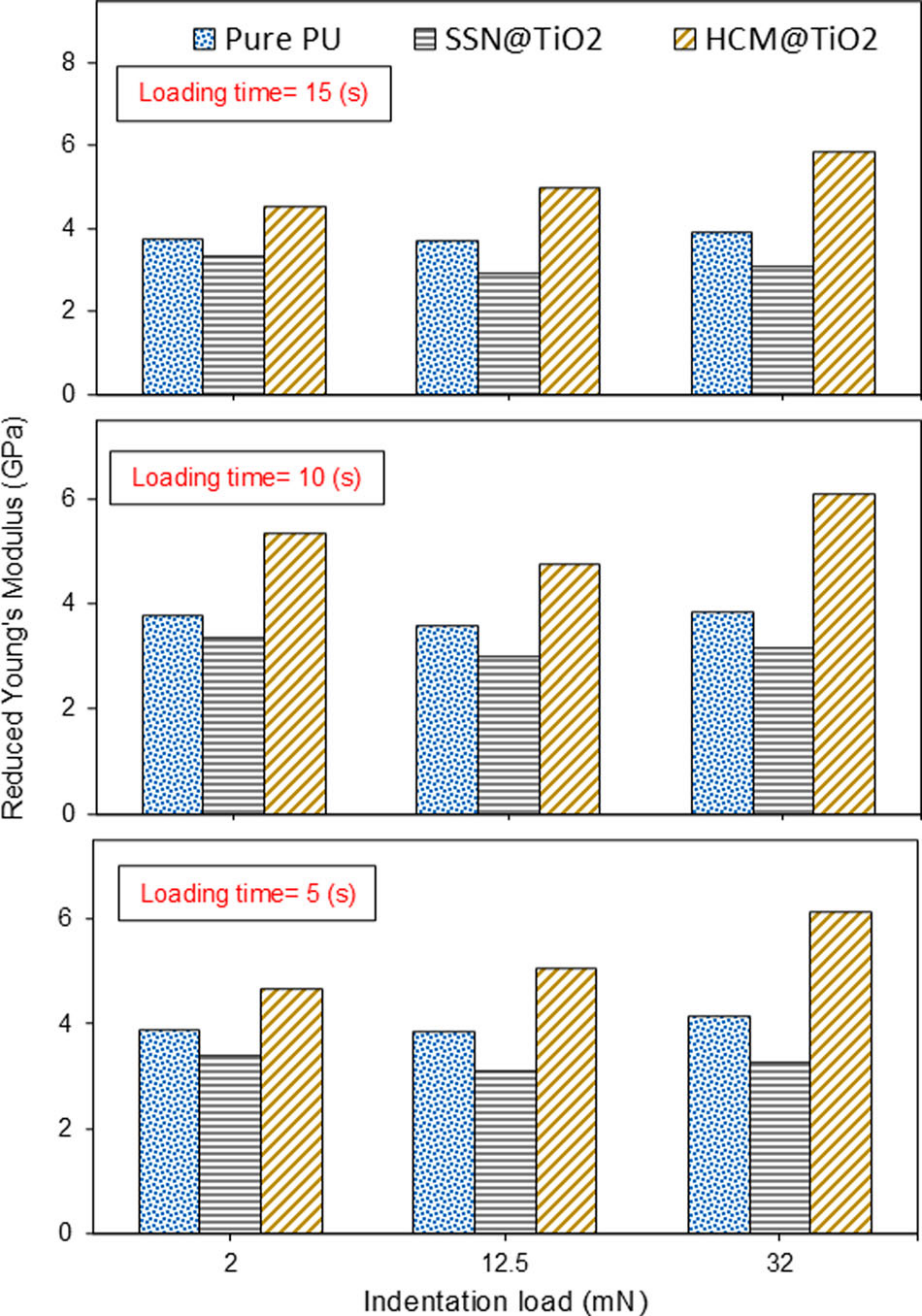
Variation in the reduced modulus as a function of indentation load and loading time.

Figure 7 presents the influence of indentation load and duration across three distinct levels on reduced modulus. *E*_*r*_ is one of the most frequently studied material properties assessed through nanoindentation testing as demonstrated by Oliver and Pharr^58^:

7 $${E}_{r}=\frac{1}{\beta }\frac{\sqrt{\pi }}{2}\frac{S}{\sqrt{{A}_{p}\left({h}_{c}\right)}}$$

where, *β* is a constant determined by the geometry of the indenter (for a Berkovich indenter it is 1.034), *S* denotes the material stiffness (derived from the slope of the unloading segment), and ${A}_{p}\left({h}_{c}\right)$ signifies the projected area of contact of the indenter.

Typically, *E*_*r*_ exhibit a direct correlation with hardness^59^ and creep resistance^60^. Consequently, the overall behaviour of this parameter aligns with the findings observed in the preceding sections. The incorporation of HCM@TiO_2_ core-shell microparticles into the Pure PU coating resulted in the increase of *E*_*r*_ value by 37.8% which was consistent with the improvement in the hardness and creep strain rate sensitivity. Furthermore, the inclusion of SSN@TiO_2_ core-shell nanoparticles led to a decrease in *E*_*r*_ by 17.1%.

Bulk modulus is another significant parameter that represents the material’s resistance to compression, and in this way, it can elucidate the deformation and creep mechanisms of various coatings. In polymers, it denotes the material’s endurance to volumetric changes under external stresses. Typically, a higher bulk modulus indicates greater resistance to creep, due to less volumetric change over time^61^. This parameter can be calculated from Young’s modulus as follows:

8 $$K=\frac{{E}_{s}}{3\left(1-2{\nu }_{s}\right)}$$

where, where ${E}_{s}$ and ${\nu }_{s}$ are the Young’s modulus and Poisson’s ratio of the material, respectively. ${E}_{s}$ can be deduced from the reduced Young’s modulus (*Er* shown in Eq. (7)), and it can be expressed as^62^:

9 $$\frac{1}{{E}_{r}}=\frac{1-{{\nu }_{i}}^{2}}{{E}_{i}}+\frac{1-{{\nu }_{s}}^{2}}{{E}_{s}}$$

where ${E}_{i}$ and ${\nu }_{i}$ represent the Young’s modulus and Poisson’s ratio of the indenter, respectively (1140 GPa and 0.07)^63^. Moreover, for the polymer coatings examined in this study, the Poisson’s ratio was assumed to be 0.40^64^. By combining the two aforementioned equations, the Bulk modulus formula can be rewritten as follows:

10 $$K=\frac{{E}_{i}{\,E}_{r}\left(1-{{\nu }_{s}}^{2}\right)}{3\left(1-{\nu }_{s}\right)\left({E}_{i}-{E}_{r}+{E}_{r}{{\nu }_{i}}^{2}\right)}$$

**Fig. 8:**
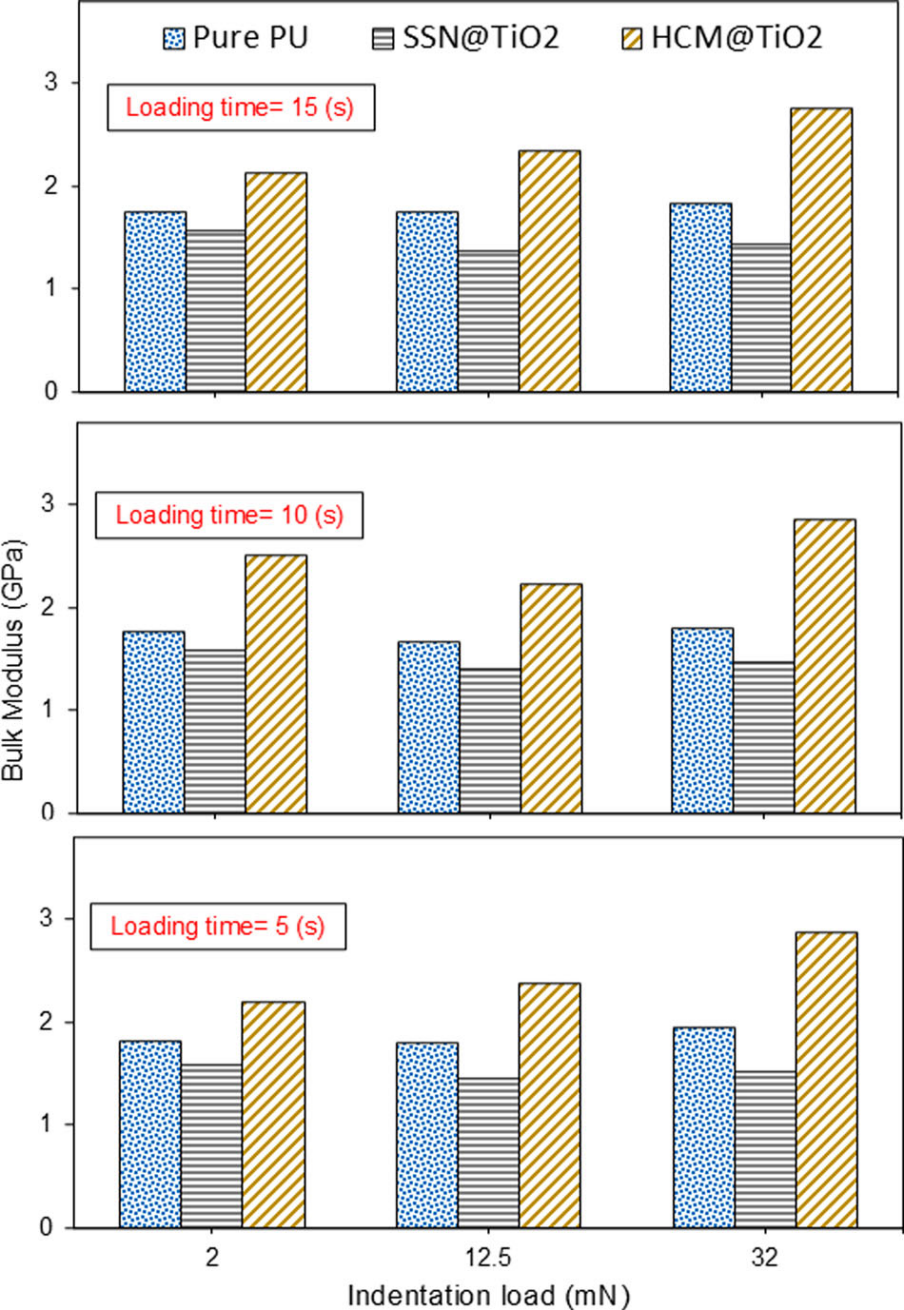
Variation of Bulk modulus as a function of indentation load and time.

Fig. 8 indicates the changes in bulk modulus across various indentation loads and durations. Since *K* is a function of *E*_*r*_, the overall trend of Bulk modulus follows identical behaviour as reduced Young’s modulus.

## Discussion

Some studies have explored the effects of incorporating SiO₂, TiO₂, and other core-shell micro- and nanoparticles into polymer substrates. Malaki et al.^65^ incorporated 4 wt% nano-silica into polyurethane substrates and observed 78% modification in its microhardness. In another work by Rajabimashhadi et al.^17^, seven combinations of various micro and nanoparticles (including SiO_2_) were used as reinforcement of PU substrate, and the best hardness improvement observed was 69%. In a recent study by Tripathi et al.^66^, the in-situ incorporation of silica (SiO_2_) microparticles into a polyurethane PU was applied to enhance its properties. While this approach led to an improved tensile strength by 83%, the improvement in hardness was merely 12.3%.

Some researchers have also investigated the incorporation of TiO_2_ particles into polymer substrates, evaluating their impact on mechanical properties. Their findings demonstrated an improvement in hardness of up to ~30% and an enhancement in creep resistance of up to ~85%^67–69^. In a recent study by Sohrabi-Kashani et al.^70^, the mechanical properties of pure polyurethane (PU) filled with nanoparticles and PU co-filled with both nano- and microparticles were compared. The results showed that increasing the titania content from 0 to 3 wt% led to a 33% improvement in hardness. However, the study also indicated that if the titania content exceeds 4%, it could interfere with the crosslinking between the polymer chains, resulting in a softer surface and reduced scratch hardness. Few studies can be found to explore the mechanical properties of polymer incorporated with core-shell particles that have shown a modified tensile and impact strength up to ~30%^71–73^.

Ultimately, considering all the aforementioned literature, it can be observed that the hardness of the novel HCM@TiO_2_ coating in this study exhibited a 111% increase compared to the pure PU substrate, which is significantly higher than that incorporation of its individual components (SiO_2_ and TiO_2_) or other investigated core-shell composites in PU.

The novel aspects discovered in this work underscored the efficacy of HCM@TiO_2_ in enhancing the creep resistance of the polyurethane coating. However, the incorporation of nano core-shell functional particles (SSN@TiO_2_) exhibited minimal effect. One potential explanation for this could be the heightened likelihood of nanoscale particle agglomeration on the polyurethane coatings^74^.

Previous studies have convincingly demonstrated the effectiveness of incorporating core-shell nanospheres to enhance various properties of polymers. These enhancements encompass increased hardness^75^, augmented resistance to crack initiation^76^, reduction in the coefficient of thermal expansion (CTE)^77^, and notable improvement in their creep behaviour^78^. However, it has also been demonstrated that nanoparticles are more prone to agglomeration compared to microparticles^74^. One primary factor contributing to this phenomenon is the reduced particle size, leading to a decrease in interparticle distance and subsequently increase in the likelihood of aggregation. These agglomerated clusters possess higher hardness than the Pure PU substrate and can act as sites for stress concentration, thus adversely affecting polymer properties. Therefore, in comparing HCM@TiO_2_ (with micro size) and SSN@TiO_2_ (with nano size), the smaller size of SSN@TiO_2_ particles leads to diminished interparticle distances, thereby increasing the likelihood of agglomeration and exacerbating stress concentration.

**Fig. 9:**
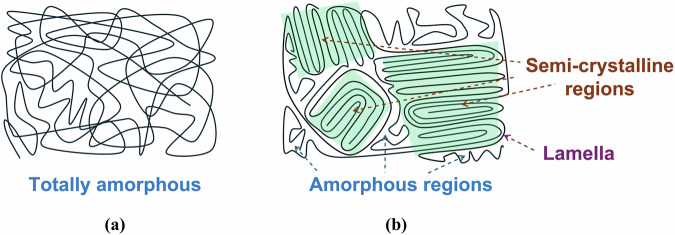
Schematic representaion of polymer chain configurations. **a** totally amorphous polymer chains, **b** polymer chains with semi-crystalline regions.

On the other hand, the structure of polymer chains could play a crucial role in determining their mechanical and creep behaviour. Typically, PU chains exhibit an amorphous structure (see Fig. 9a)^79^. However, under specific conditions, they can orient themselves into parallel configurations, forming a semi-crystalline structure, as illustrated in Fig. 9b. Infusing micro- and nanoparticles into the polymer substrate is a strategy for attaining the lamellar structure^80–82^. The emergence of new spectral ranges in HCM@TiO_2_ core-shell particles can be seen from the FTIR results that were shown earlier in Fig. 2. Apart from that, increased crystallinity in polymers correlates with a higher glass transition temperature (T_g_)^83^, which is one of the main factors contributing to the improved creep resistance^84^.

**Fig. 10:**
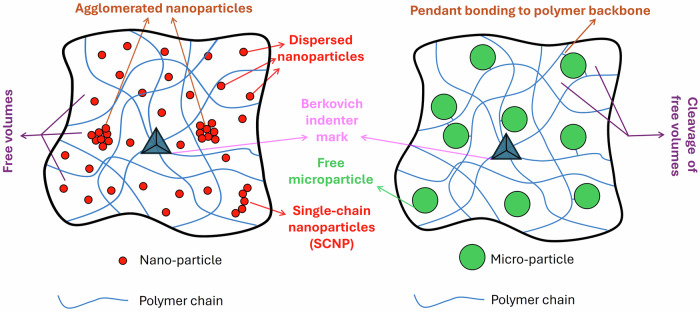
Effect of nano- and microparticles on polymer chain movement and free volumes.

The free volume (FV) concept is another crucial aspect that determines the behaviour of polymers. As depicted in Fig. 10, FVs are delineated as the interstitial spaces or pores existing between polymer chains, devoid of basic material occupancy. FVs (and consequently, the polymer properties) can be influenced by temperature^85^, pressure^86^, atmosphere^87^, and more importantly, additive particles^88^. Previous studies have indicated that some nanoparticles, such as nano-SiO_2_^89^, nano-TiO_2_^90^, and nano-ZnO^91^, have the potential to act as plasticisers, thereby diminishing the strength of polymers. Plasticisers are liquid or solid additive particles that are added to the polymers and make them more flexible. Accordingly, the presence of nanoparticles could promote polymer chain sliding, consequently diminishing its resistance to creep. In contrast, the hollow SSN@TiO_2_ core-shells can serve as polymer fillers, effectively acting as barriers to restrict the movement of FVs. They also shrink the free volumes, serving as a method to enhance the mechanical properties of polyurethane^92^.

Furthermore, the additives can interconnect polymer chains (as schematically shown in Fig. 10), thereby augmenting the intermolecular forces between them^93^. In such a way, the fragmentation of free volumes results in smaller entities. Given that decreasing the size of free volumes typically corresponds to improved mechanical properties, this phenomenon provides another rationale for the enhanced creep resistance observed in HCM@TiO_2_ core-shell coatings. Besides, incorporating additives and hard particles into polymers can restrict the movement and deformation of molecular chains, thereby improving mechanical and creep behaviour^94,95^. From this perspective, particle size and shape, along with dispersion and interaction with the polymer matrix, could affect the polymer strength. However, this could be trick,y and there are trade-offs between these parameters and polymeric strength. Previous investigations demonstrated that at a constant particle concentration, the smaller particle size results in improved cross-linking and consequently, better mechanical properties^96^. However, excessively reducing the particle size may have the opposite effect. This was observed in the experiments where SSN@TiO₂ showed detrimental effects, likely due to their role as plasticisers and the increased potential for agglomeration resulting from their higher surface energy.

On the other hand, the morphology of these hard particles plays a significant role and could influence both the rheology of polymer network^97^ and its strength^98^. Although other particle forms, such as nanowires, nanotubes, and mesoporous structures, could also be considered, the specific focus of this work was on the spherical shape of micro/nano additives. Their symmetrical geometry enables more even stress distribution within the polymer, minimising stress concentration points that may arise from irregular shapes^99^.

Another aspect of interest to note here was that the micron-sized SiO_2_ particles that were used to prepare HCM@TiO_2_ were hollow inside, which makes them light-weight, and by weight they measure the same as the solid nanoparticles. It suggests that the right design-to-manufacture strategy of the core-shell nanoparticles can provide a new avalanche of opportunities to design creep and fatigue-resistant polymeric coatings for critical safety applications.

Overall, the key findings obtained in this investigation can be summarised as:

- The PU coating incorporating hollow ceramic microspheres with a TiO_2_ shell (HCM@TiO_2_) demonstrated superior mechanical properties compared to coatings with nanosized solid silica core-shells, achieving unprecedented improvements in creep behaviour.
- Typically, increasing indentation load results in reduced creep strength. However, the HCM@TiO_2_-incorporated PU coating exhibited a reverse trend, which could be advantageous under incremental loading conditions.
- The PU coating incorporating solid silica nanospheres with a TiO_2_ shell (SSN@TiO_2_) showed significant deterioration in both mechanical and creep properties. This degradation, observed across various loading conditions, emphasized the limitations of nanosized additives in this context.
- SSN@TiO_2_ core-shell nanoparticles acted as plasticizers, leading to an increased agglomeration within the PU matrix. In contrast, HCM@TiO_2_ microparticles enhanced the crystallinity of the PU and reduced free volume by forming pendant bonds with the polymer backbone. These mechanisms, tied to the use of micron-sized additives, were identified as the key factors behind the improved creep performance of the HCM@TiO_2_ coating.

Future research in this area will prioritize detailed microstructural characterization of the HCM@TiO_2_-blended PU coatings, with a particular emphasis on examining the interfaces and understanding how the incorporation of HCM@TiO_2_ modifies the inherent crystallinity of the PU polymer.

## Methods

### Sample preparation

The hollow ceramic microsphere (HCM) particles used in this study were obtained from SiO_2_-Al_2_O_3_ ceramic, derived as a by-product of coal-fired plants in the Donetsk and Ekibastuz coal basins. Its properties and characteristics have been extensively examined in our past investigations^100^. The ceramic’s chemical composition was (SiO_2_ - 56.5 wt.%, Al_2_O_3_ - 36.9 wt.%, CaO -2.4 wt.%, and others, including Fe_2_O_3_, MgO, TiO_2_ K_2_O, Na_2_O, less than 1 wt.%). Titanium tetra isopropoxide (TTIP) (purity 98%) and Methyl trimethoxy-silane (MTMS) were acquired from Sigma Aldrich, UK. HNO_3_, NH_4_OH, and ethanol were sourced from Merck, UK. The transparent polyurethane media was procured from Dalton Chemicals Pvt. Limited, India.

For the synthesis of HCM@TiO_2_ core-shell microspheres, we utilised the sol-gel method^28^. In brief, the core-shell structure was developed by adding 5 mL of TTIP precursor into a 1:1 mixture of isopropyl alcohol and distilled water containing 2 grams of HCM, with continuous stirring. After 20 min of stirring, NH_4_OH was added, and the solution was stirred for an additional 40 min. A white precipitate was separated from the medium by centrifugation at 8000 rpm for 20 min. The precipitate was then washed with ethanol and acetone and dried overnight at 100 °C. Finally, the sample was calcinated at 500 °C for 4 h to produce the core-shell nanoparticles.

To ensure robust adhesion, the synthesised core-shell microparticles were functionalised using MTMS. In this trial, a solution of 20 mL ethanol was employed to dissolve 1 gram of HCM@TiO_2_ CS, which was then blended with 0.2 mL of MTMS and subjected to continuous stirring for 4 h. Furthermore, 1 mL of NH_4_OH was slowly introduced into the mixture. The sample underwent heating for a duration of 20 to 30 min at 70 °C to facilitate the evaporation of ethanol, resulting in the production of functionalised micro core-shell particles. The method to prepare SSN@TiO_2_ core-shell particles has already been reported in our previous works^54^.

The HCM@TiO_2_-infused PU coatings were crafted through a process involving the amalgamation of PU binder, thinner, and HCM@TiO_2_ core-shell particles using probe sonication. Maintaining a fixed binder-to-thinner ratio of 1:2 in the formulation, the study found the optimal loading percentage of HCM@TiO_2_ CS for peak performance by adjusting its mix ratio. The coating formulations ranged from 0.5% to 6% (wt.) microparticles, allowing for various proportions to be evaluated. To ensure a uniform distribution of additive particles, the polymer coating and an ultrasonic homogeniser were employed for a sufficient duration just before application on the substrate. Using a paintbrush, coatings of different concentrations (0.5% to 6% wt.) were applied onto low carbon steel substrates (Grade: 1006-1026^101^) (2.5 × 2 cm^2^) and air-dried for an hour, resulting in a fully dried, nearly uniform coating with a thickness of about 98 ± 10 µm.

### Sample characterisation using SEM, XRD, and FTIR

The morphology of HCM and HCM@TiO_2_ was analysed using high-resolution secondary electron SEM imaging with Zeiss SUPRA 55VP scanning electron microscopy. A Bruker D8 Advance was used to generate the XRD spectra of pristine HCM and HCM@TiO_2_ CS microspheres. The FTIR analysis was conducted using a Shimadzu 8400 spectrophotometer. Spectra were obtained in the transmission mode over a range of 400–4000 cm^−1^.

### Nanoindentation creep testing

**Fig. 11:**
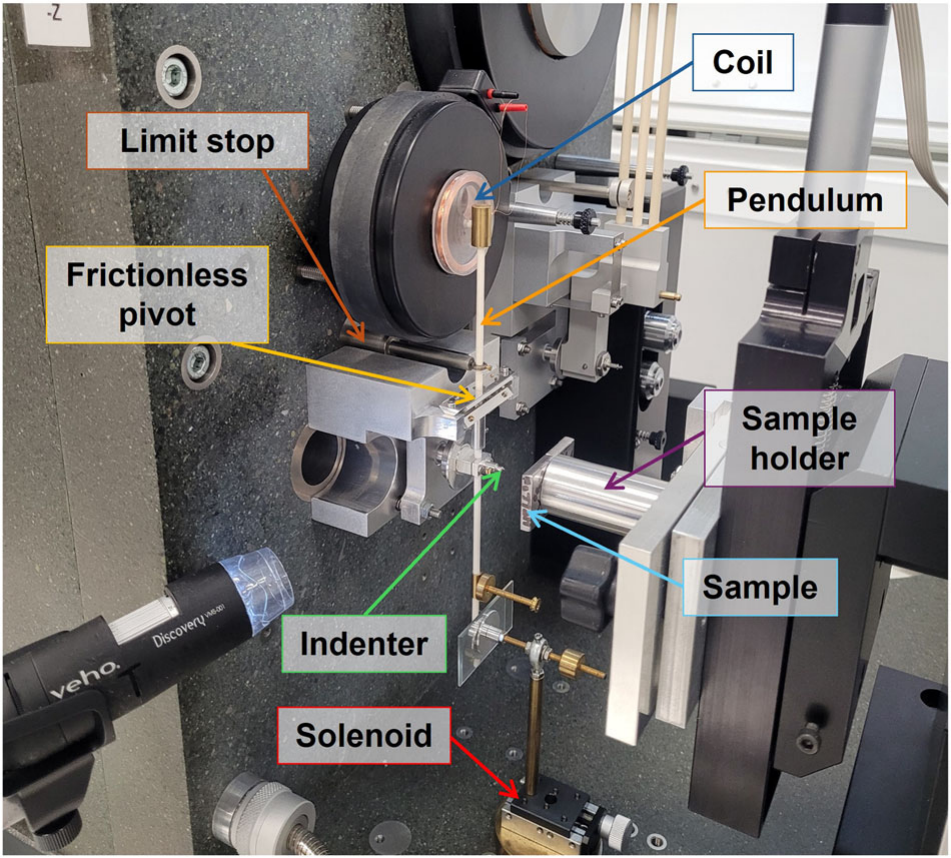
Nanoindentation setup.

A commercial nanoindentation apparatus (NanoTest Vantage (V5), Micro Materials, UK) equipped with a diamond Berkovich indenter was utilised to study the creep behaviour (Fig. 11). The nanoindentation comes equipped with a standard optical microscope and a 3D nanopositioner.

The indentation sites were randomly selected while maintaining adequate spacing to prevent interference effects, and the low standard deviation in the results confirmed the minimal influence of particle distribution on the measured properties.

**Fig. 12:**
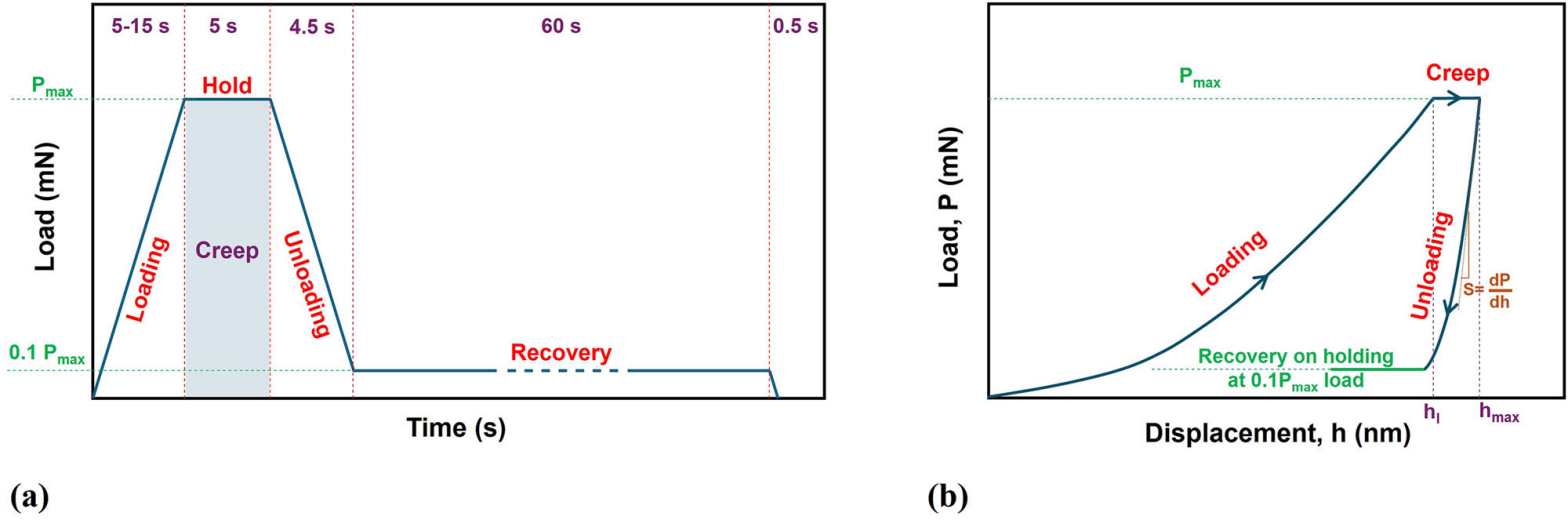
Schematic illustration of the nanoindentation test procedure and output. **a** loading function, **b** a typical nanoindentation curve.

The load function used to perform the creep test is shown in Fig. 12. It consists of three primary stages. Initially, the indenter was prescribed a linearly increasing load until a peak load. The loading times were set at 5, 10, and 15 s with peak loads of 2, 12.5, and 32 mN. These parameters were chosen following the guidelines outlined in ISO 14577, which recommends controlling the indenter’s approach speed to avoid impact effects. With the achieved indentation depths ranging from 700 to 3800 nm and loading times between 5 to 15 s, the resulting loading rates varied from 46 to 760 nm/s. These values align well with the prescribed limits specified in the standard.

Subsequently, the indenter was held at the peak load (dwell time) for 5 s to allow for the time-dependent deformation. This period depends on the material’s hardness and can vary accordingly, which is why the ISO standard does not specify a specific value. The dwell time was determined based on the preliminary experiments and the soft nature of the polyurethane substrate.

Finally, the unloading was carried out as the indenter was unloaded in 5 s. After 90% unloading, an additional hold time of 60 s was allowed to assess creep recovery and thermal drift correction. Thermal drift, caused by temperature fluctuations within the instrument, can introduce displacement errors over time, affecting measurement certainty, particularly for creep-sensitive materials^102^. Consequently, a total of 27 different runs (9 conditions for each of the 3 samples) were performed. Table 1 summarises the general conditions of these tests. For brevity, henceforth, coatings of pure polyurethane, solid silica nanosphere at TiO_2_, and hollow ceramic microsphere at TiO_2_ will be abbreviated as Pure PU, SSN@TiO_2_, and HCM@TiO_2_, respectively.

**Table 1:** Process parameters used for the nanoindentation creep tests

Nanoindentation apparatus	NanoTest Vantage V5 (Micro Materials)
Nanoindenter	Berkovich (new tip)
Base coating material	Pure Polyurethane (Pure PU)
Core-shell particles (additives mixed with PU)	Solid silica nanosphere@TiO_2_ (SSN@TiO_2_) Hollow ceramic microsphere@TiO_2_ (HCM@TiO_2_)
Peak load (P_max_)	2, 12.5, 32 mN
Loading time (T_l_)	5, 10, 15 s
Dwell period (T_d_)	5 s
Creep recovery (T_r_)	60 s
Unloading time (T_u_)	5 s

## Supplementary information

Supplementary information

## Data Availability

The datasets used and/or analysed during the current study can be accessed from: https://github.com/lsbu1/Creep/raw/refs/heads/main/Creep%20Raw%20Data.xlsx.
