# Supplementary material for: Design and development of a novel polymer coating system with exceptional creep resistance
Source: Npj Mater Sustain. 2025 Jun 30;3(1):21. doi: 10.1038/s44296-025-00063-x (PMC12208877; doi:10.1038/s44296-025-00063-x)
Supplement: Supplementary file 1 — Supplementary information [file 44296_2025_63_MOESM1_ESM.docx]

Supplementary information:

| 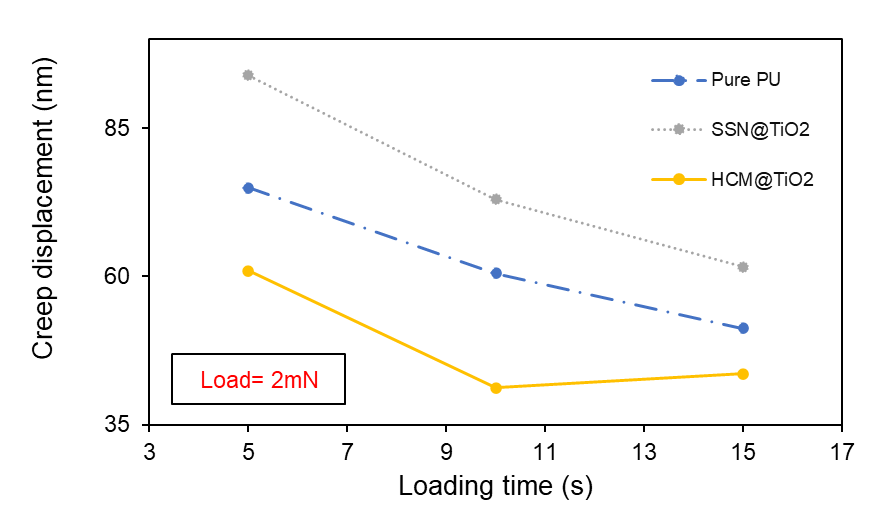 |
| --- |
| Figure S1. Creep displacement versus loading time for of indentation load 2 mN. |

| 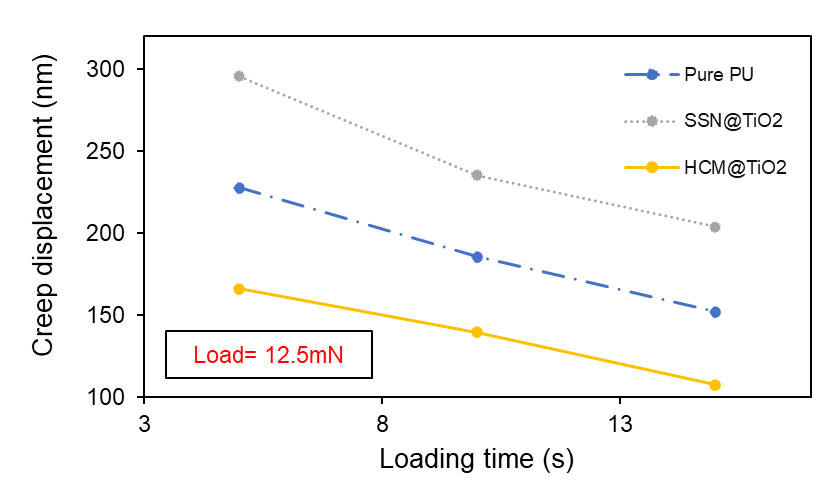 |
| --- |
| Figure S2. Creep displacement versus loading time for of indentation load 12.5 mN. |

| 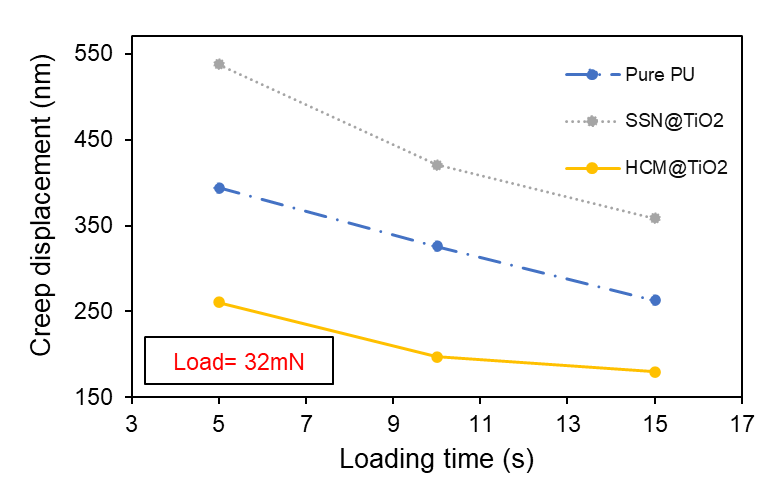 |
| --- |
| Figure S3. Creep displacement versus loading time for of indentation load 32 mN. |

| 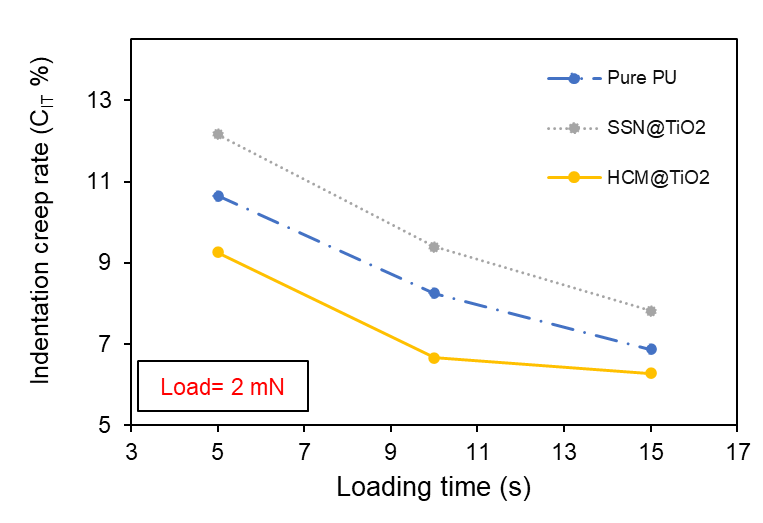 |
| --- |
| Figure S4. Indentation creep rate versus loading time for indentation load of 2 mN. |

| 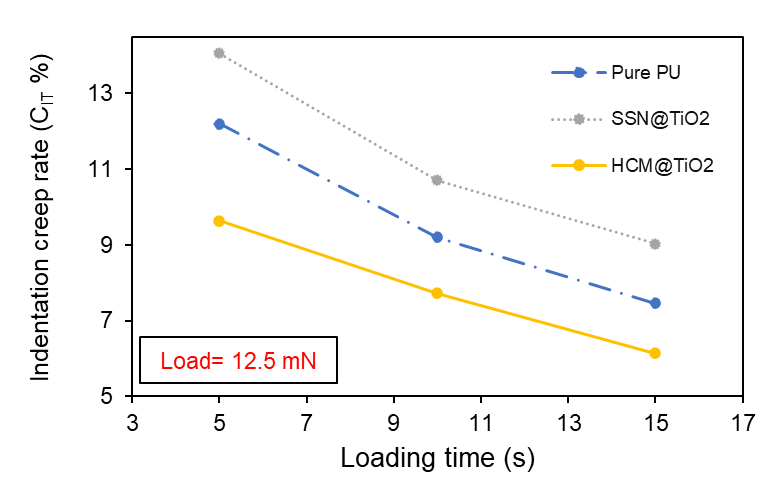 |
| --- |
| Figure S5. Indentation creep rate versus loading time for indentation load of 12.5 mN. |

| 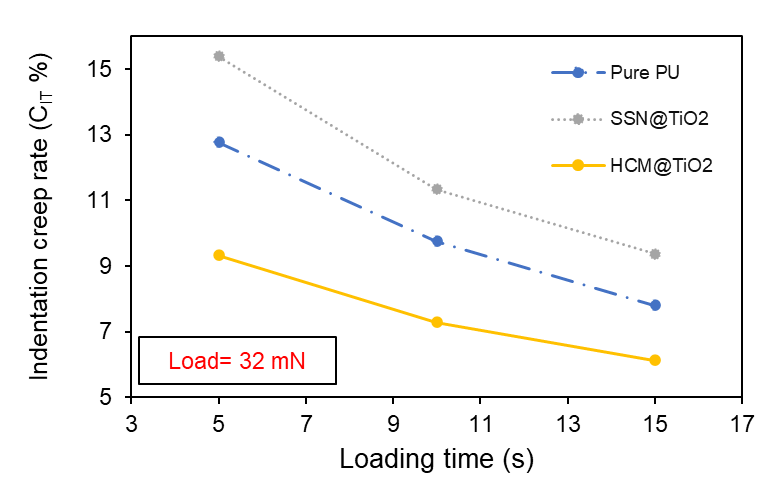 |
| --- |
| Figure S6. Indentation creep rate versus loading time for indentation load of 32 mN. |

| 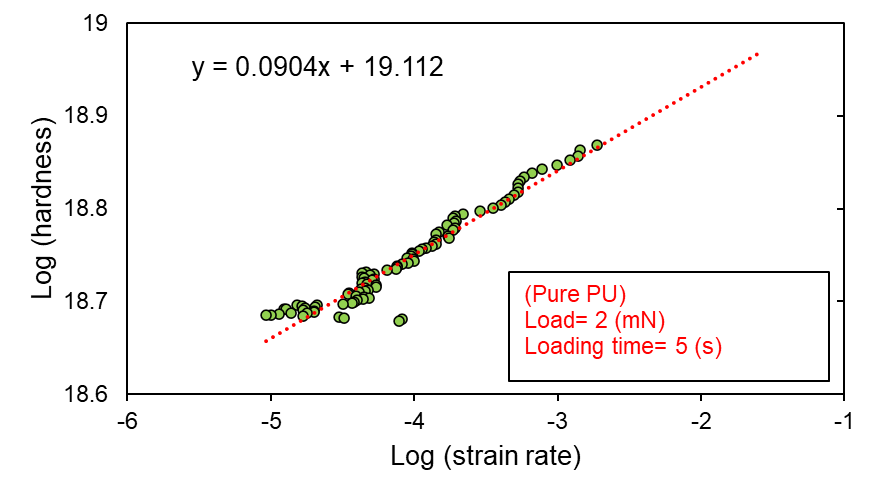 |
| --- |
| 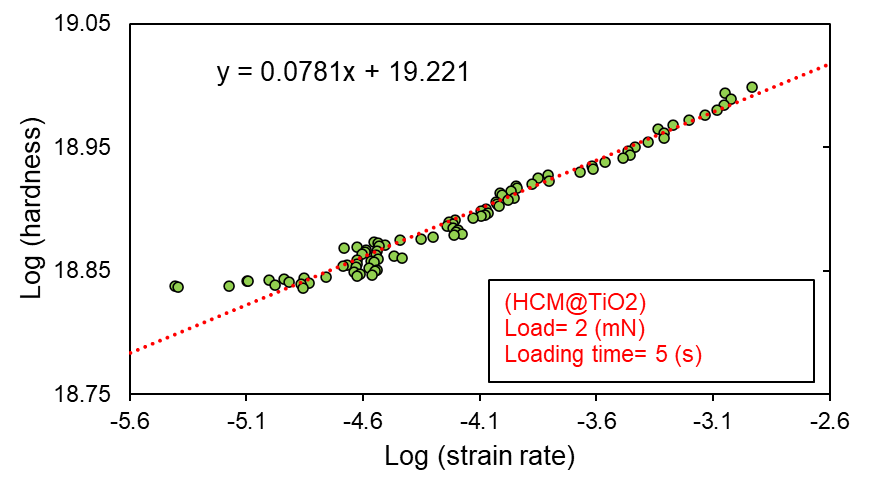 |
| 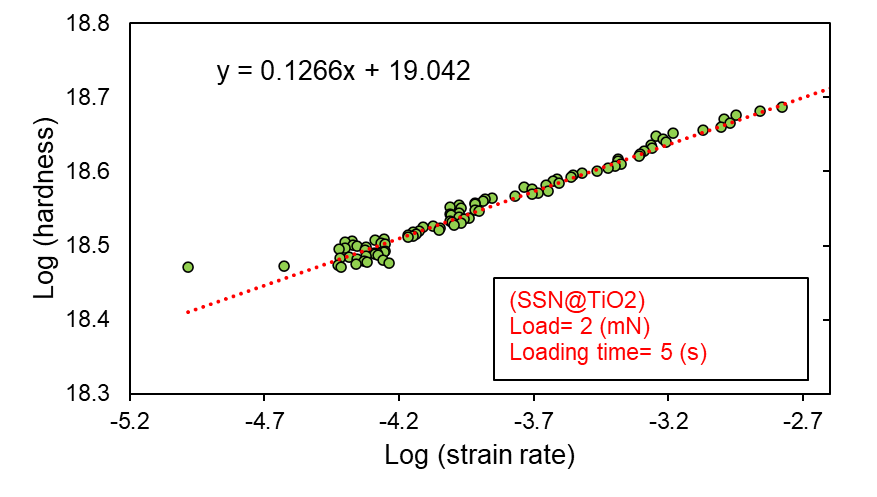 |
| Figure S7. Log (hardness) versus Log (strain rate) of different coatings under the load of 2 mN and loading time of 5 s. |

| 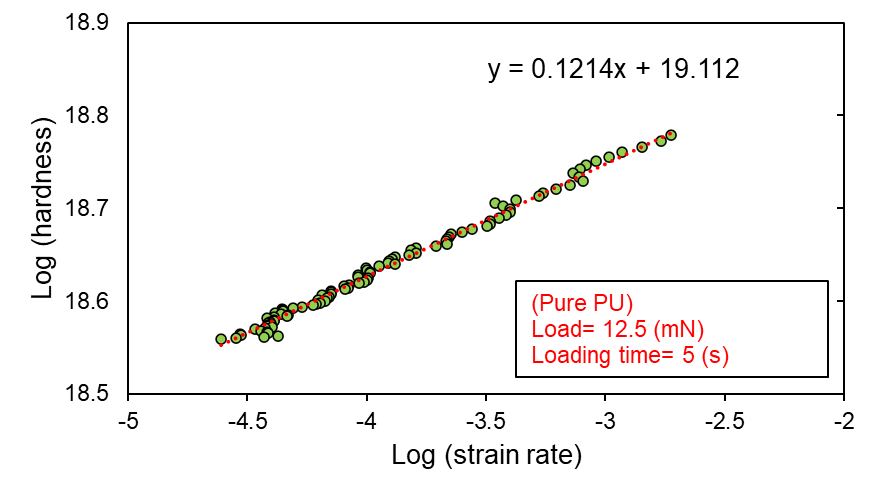 |
| --- |
| 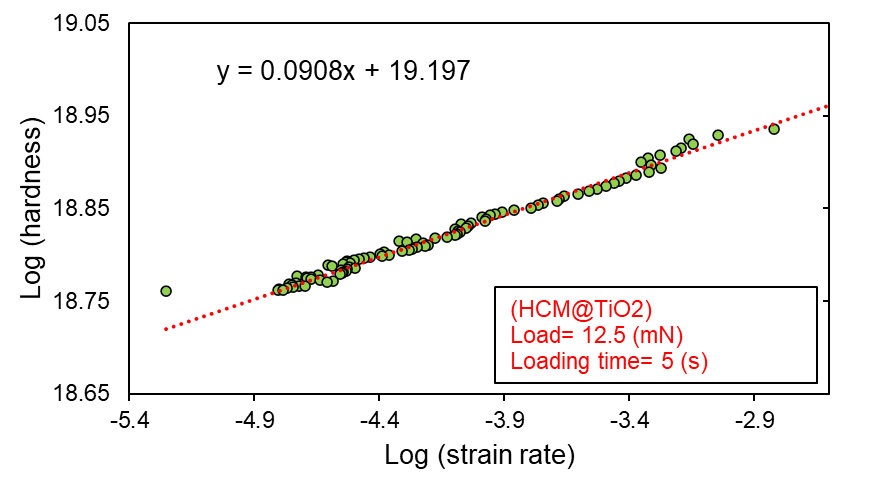 |
| 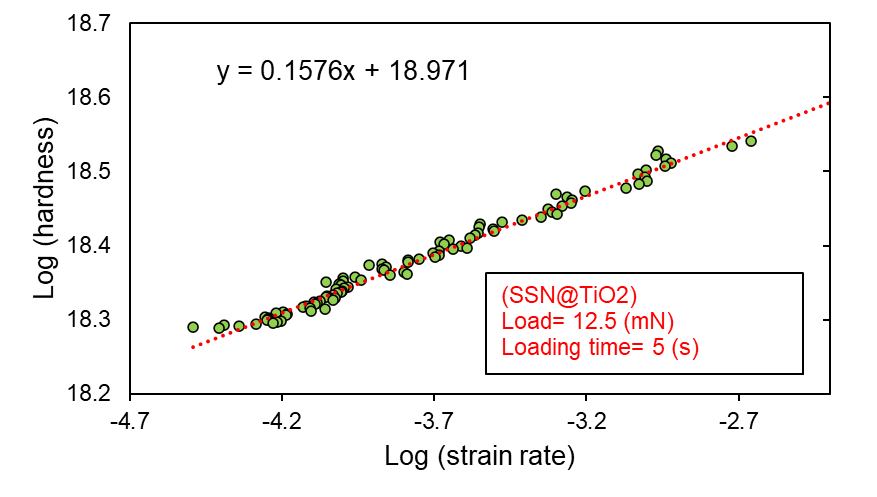 |
| Figure S8. Log (hardness) versus Log (strain rate) of different coatings under the load of 12.5 mN and loading time of 5 s. |

| 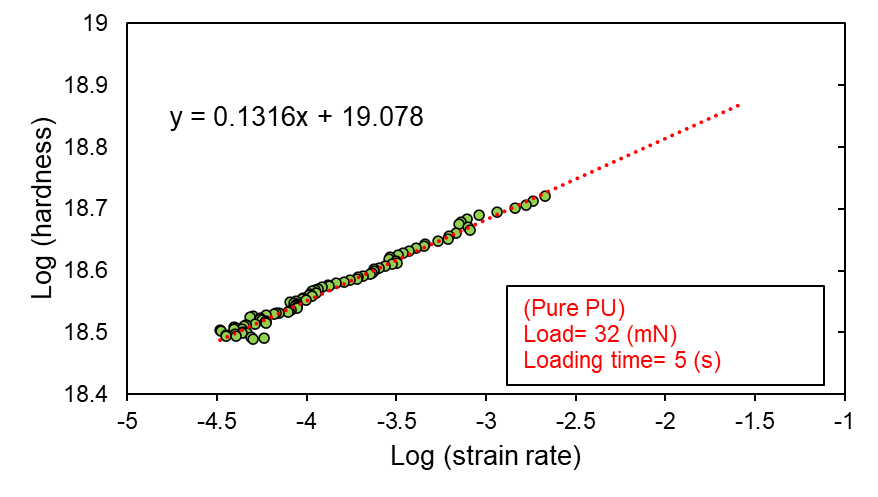 |
| --- |
| 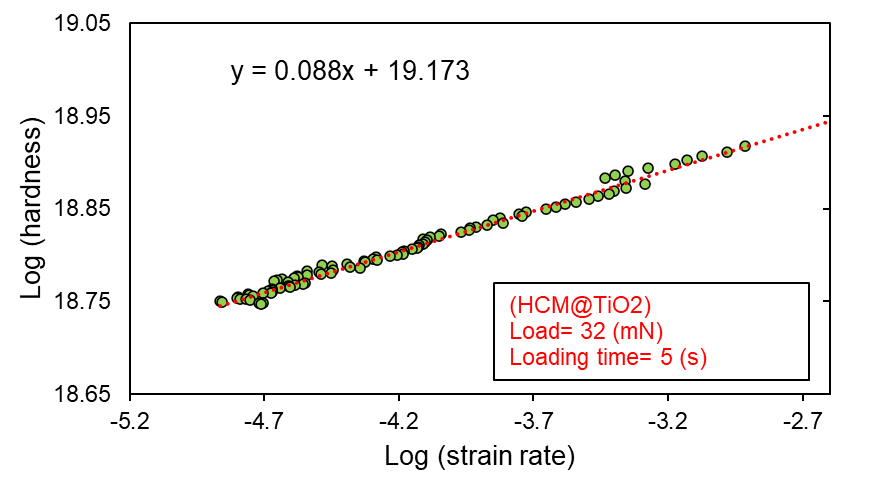 |
| 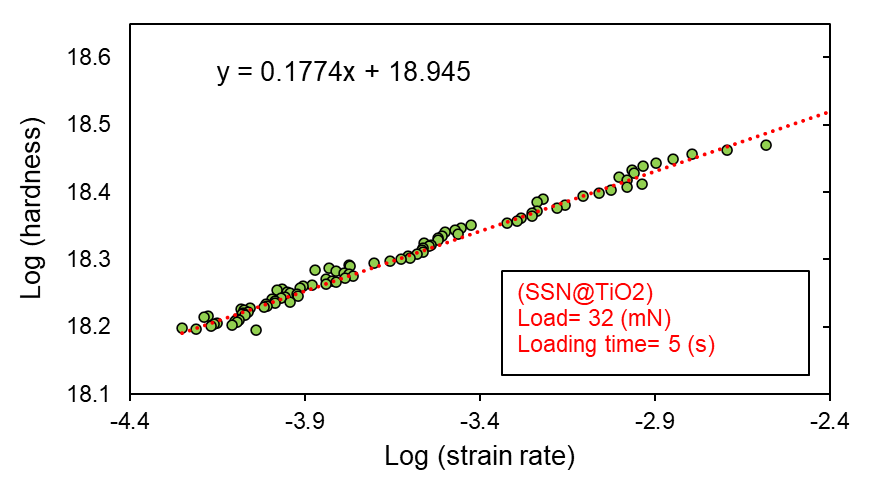 |
| Figure S9. Log (hardness) versus Log (strain rate) of different coatings under the load of 32 mN and loading time of 5 s. |

| 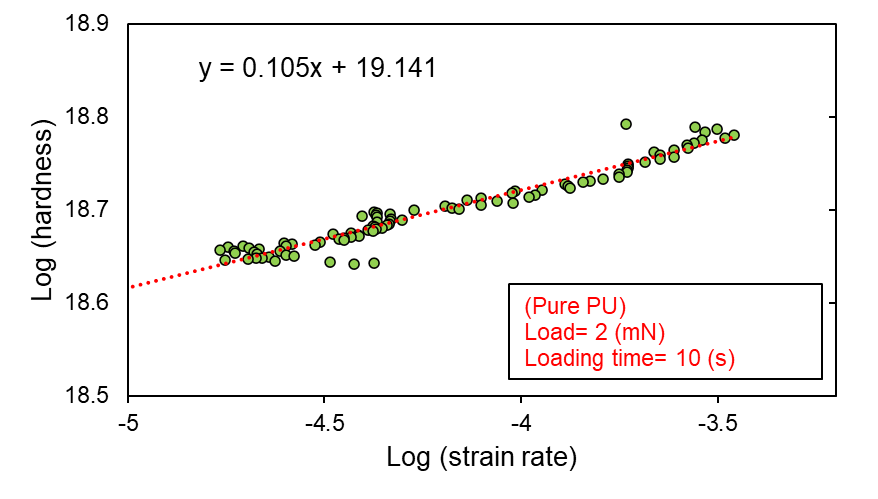 |
| --- |
| 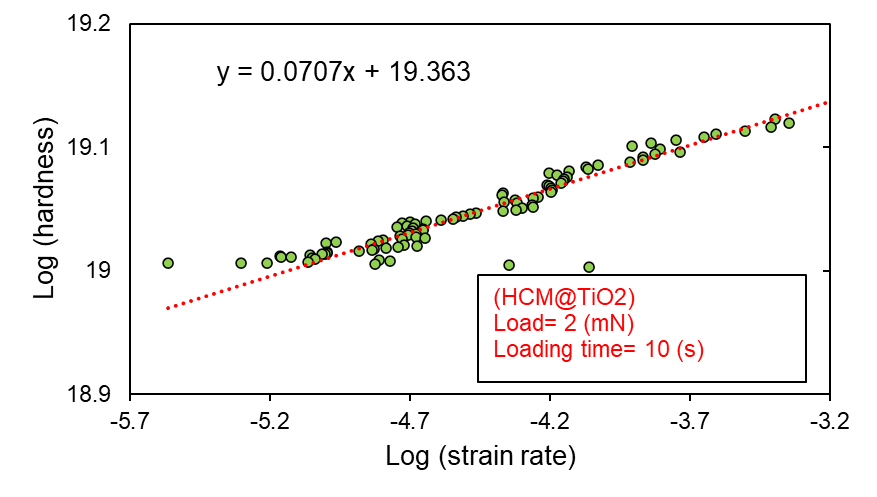 |
| 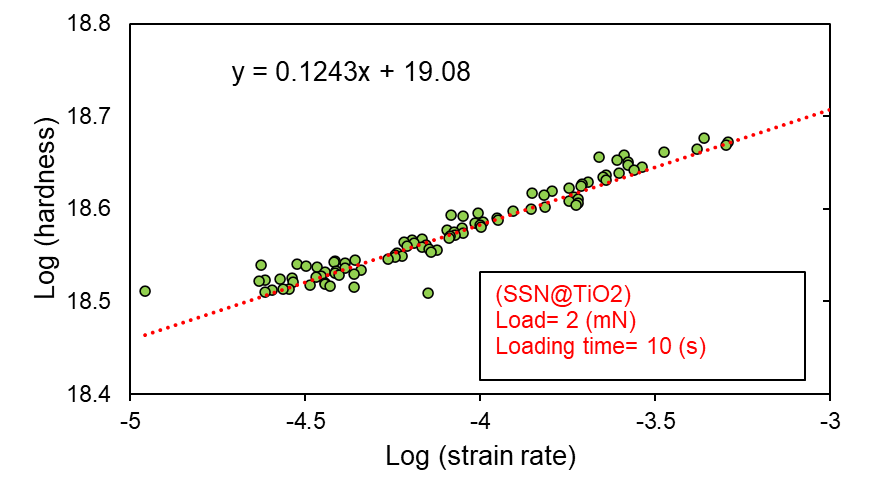 |
| Figure S10. Log (hardness) versus Log (strain rate) of different coatings under the load of 2 mN and loading time of 10 s. |

| 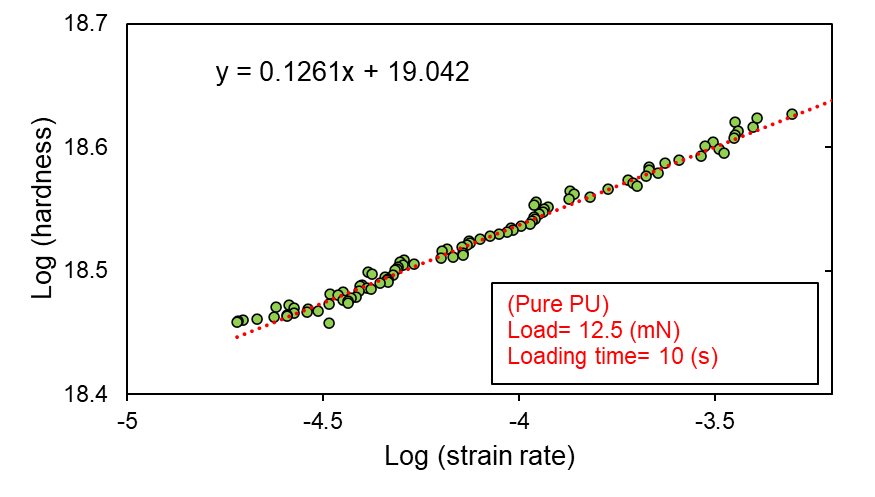 |
| --- |
| 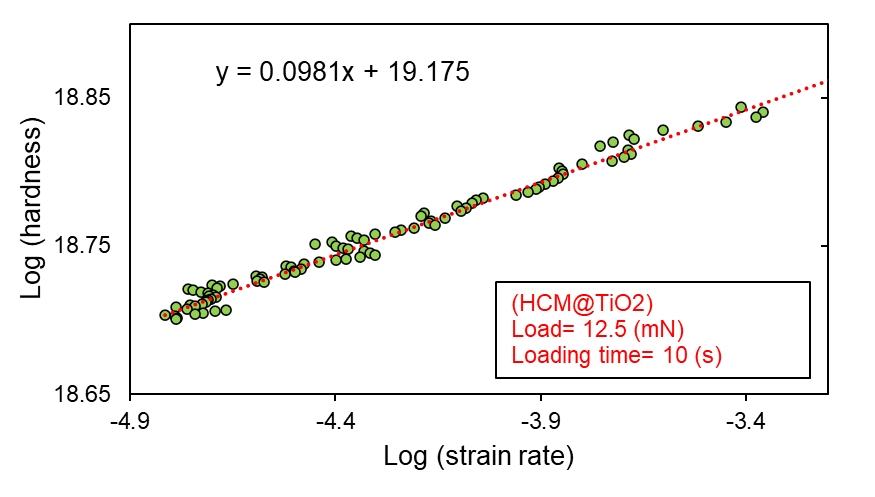 |
| 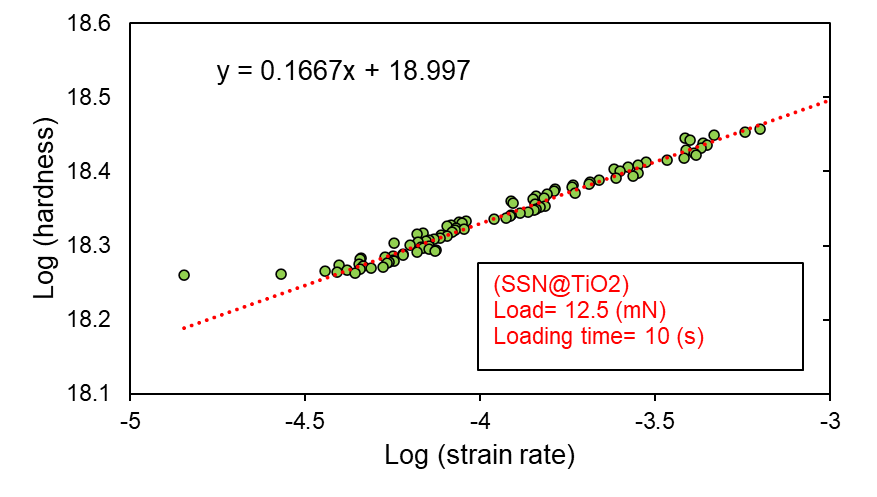 |
| Figure S11. Log (hardness) versus Log (strain rate) of different coatings under the load of 12.5 mN and loading time of 10 s. |

| 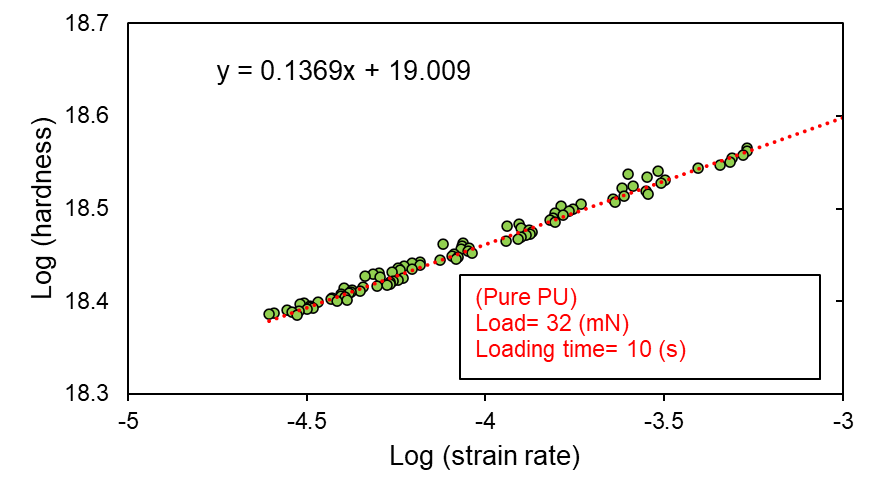 |
| --- |
| 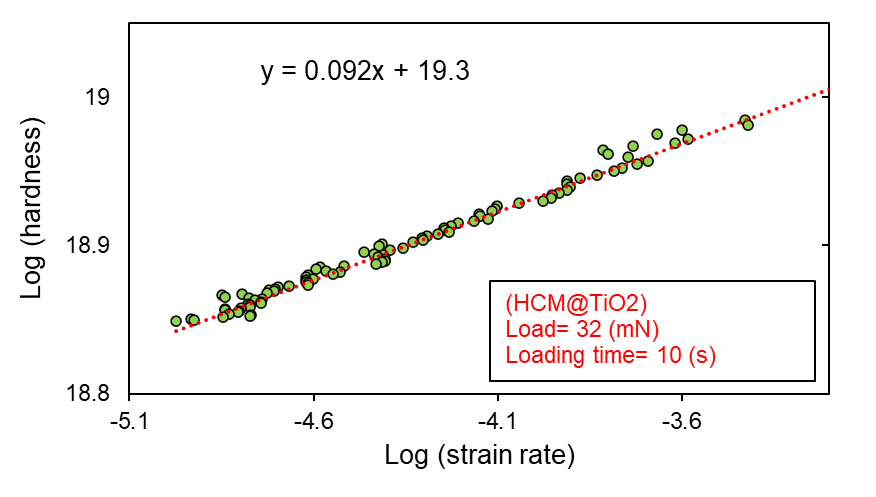 |
| 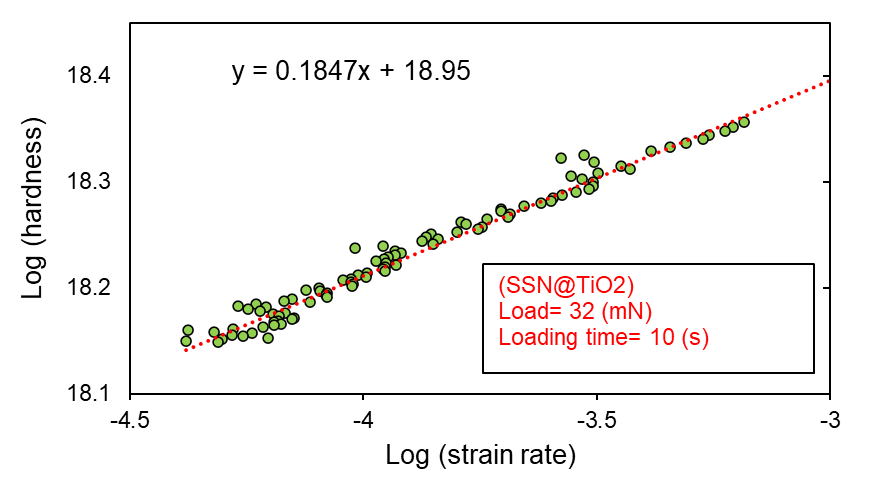 |
| Figure S12. Log (hardness) versus Log (strain rate) of different coatings under the load of 32 mN and loading time of 10 s. |

| 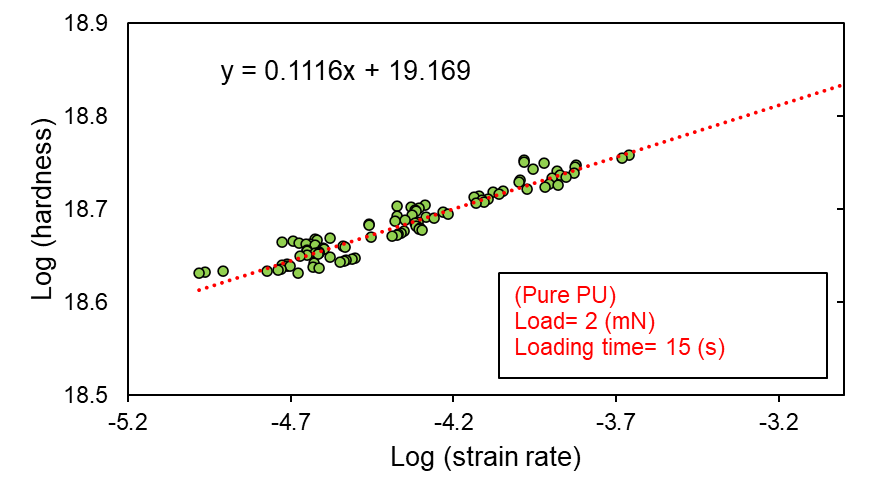 |
| --- |
| 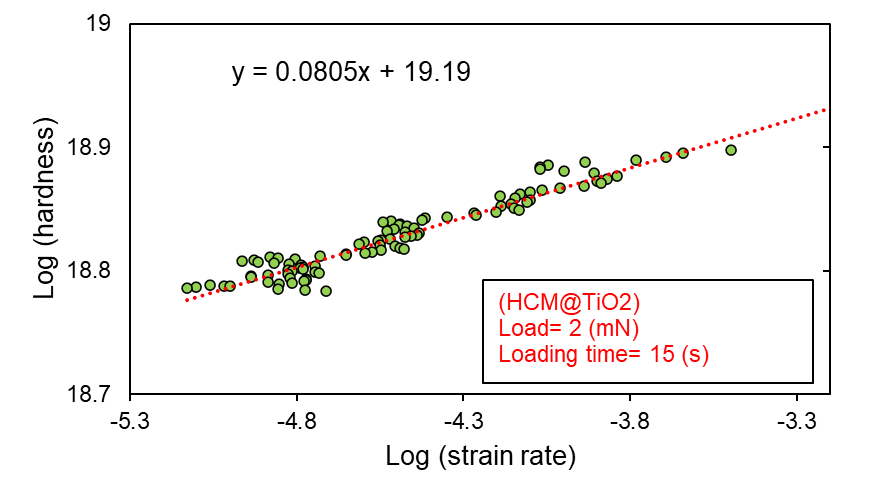 |
| 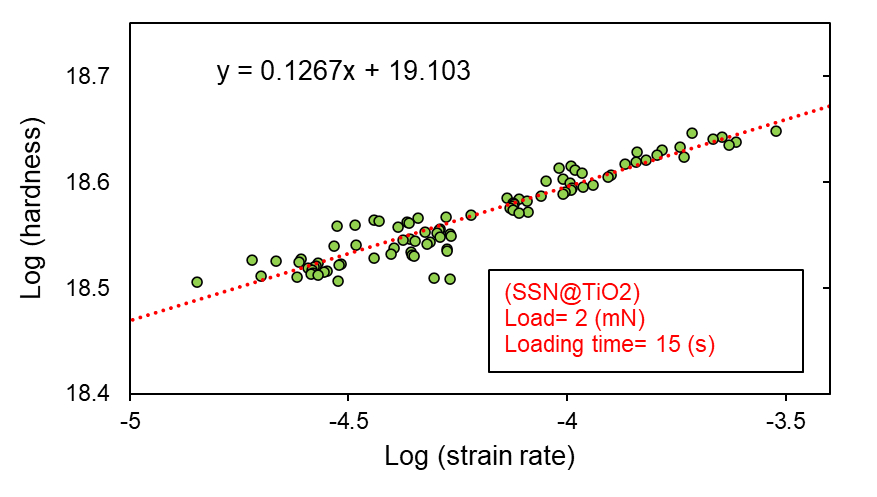 |
| Figure S13. Log (hardness) versus Log (strain rate) of different coatings under the load of 2 mN and loading time of 15 s. |

| 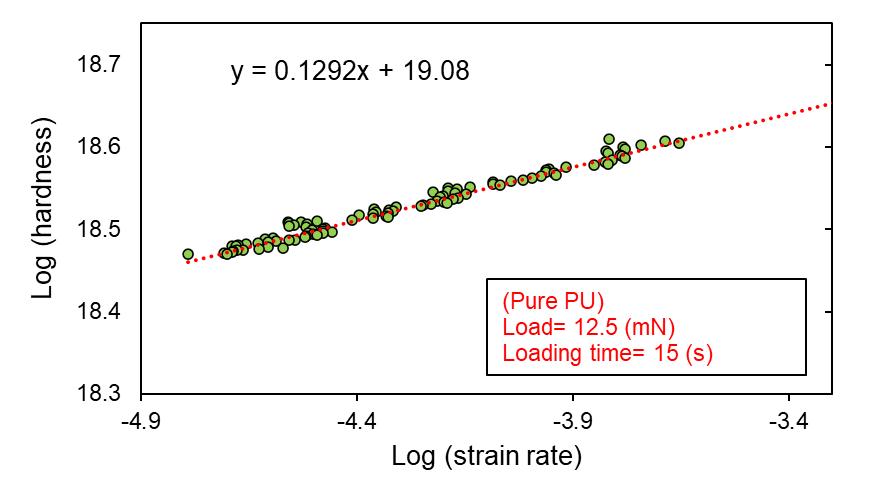 |
| --- |
| 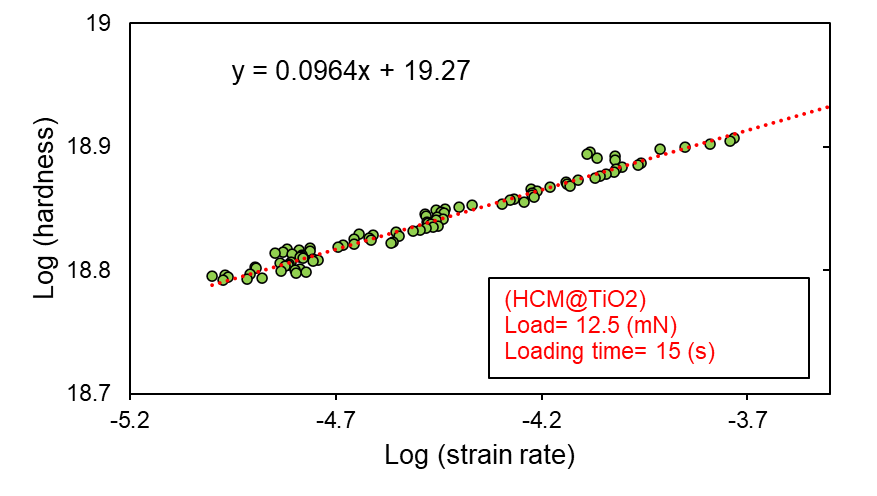 |
| 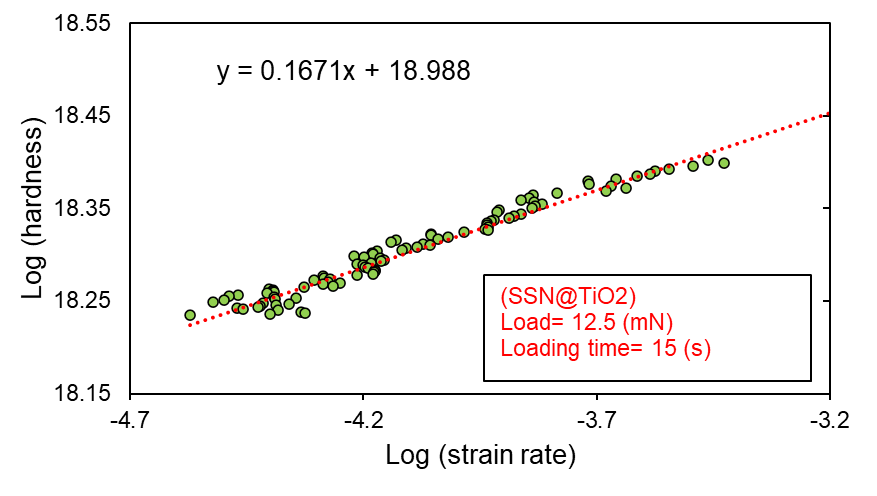 |
| Figure S14. Log (hardness) versus Log (strain rate) of different coatings under the load of 12.5 mN and loading time of 15 s. |

| 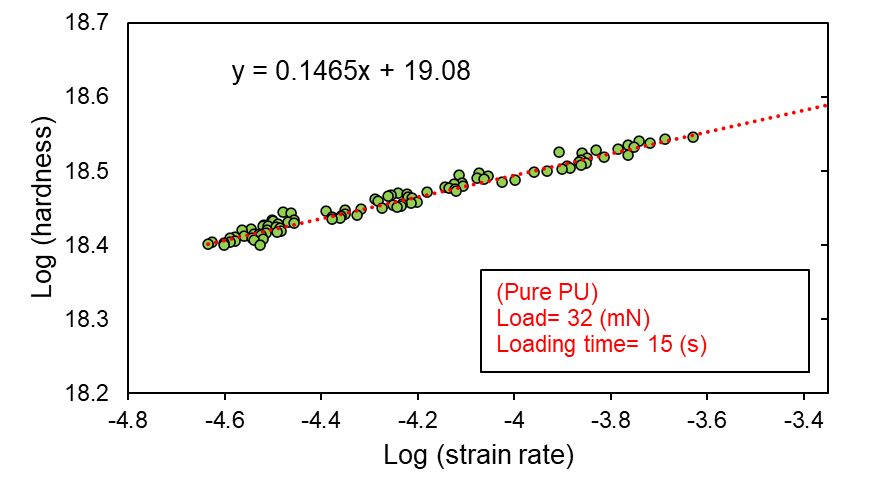 |
| --- |
| 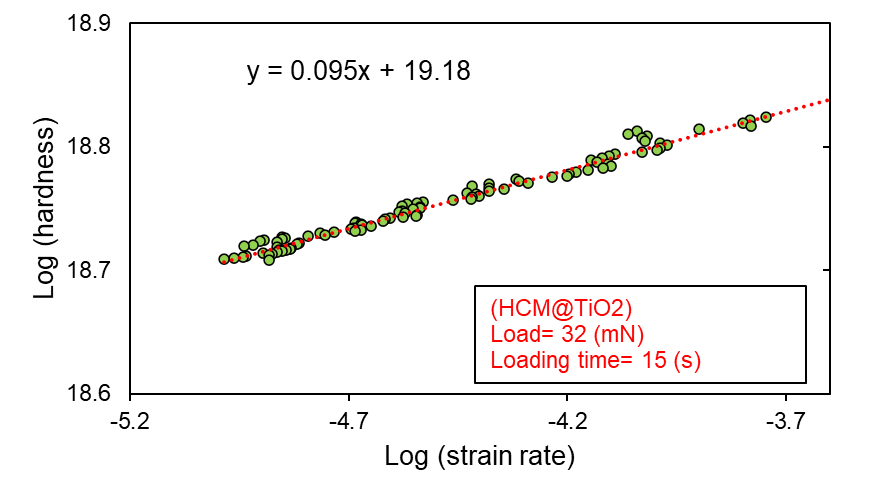 |
| 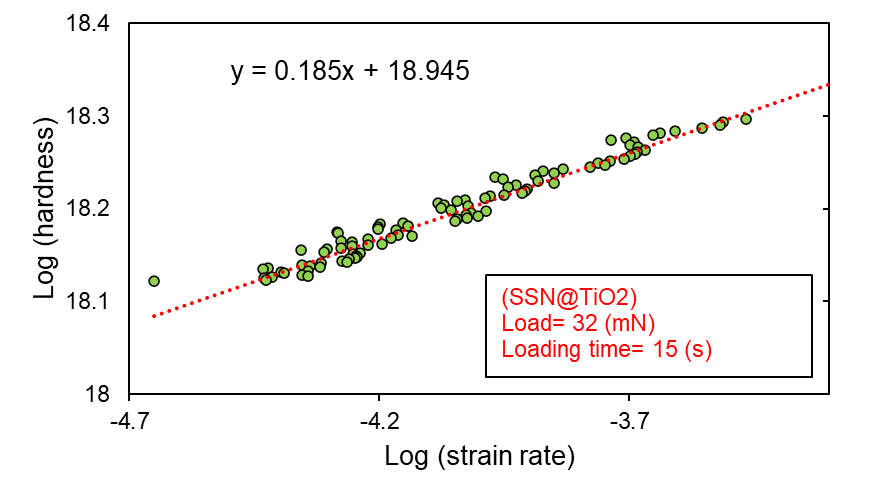 |
| Figure S15. Log (hardness) versus Log (strain rate) of different coatings under the load of 32 mN and loading time of 15 s. |

| **(a)** | 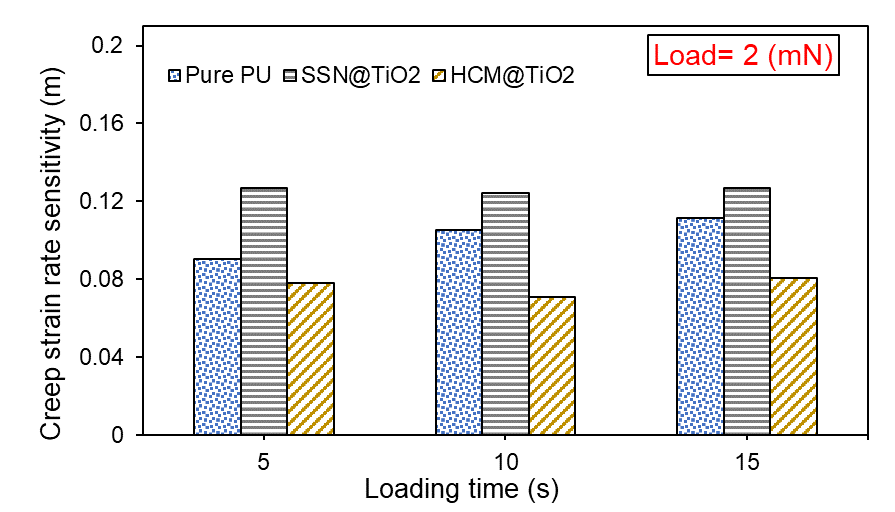 |
| --- | --- |
| **(b)** | 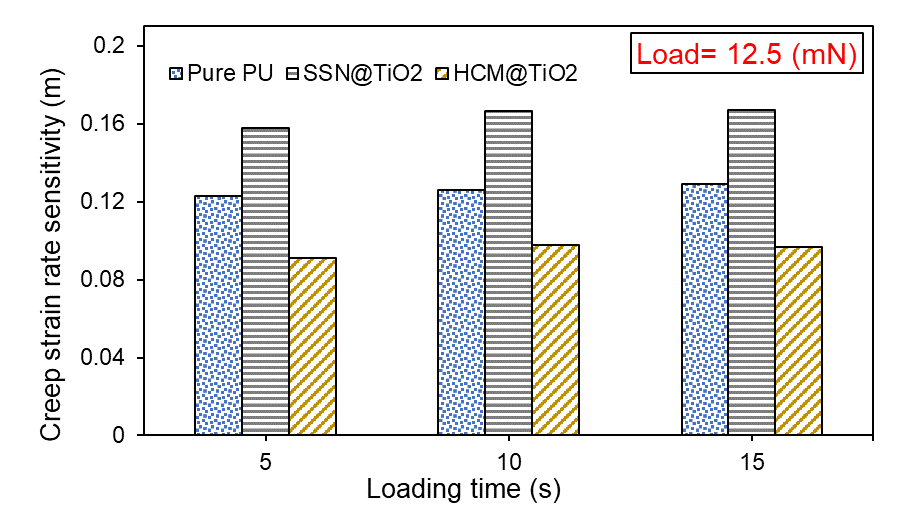 |
| **(c)** | 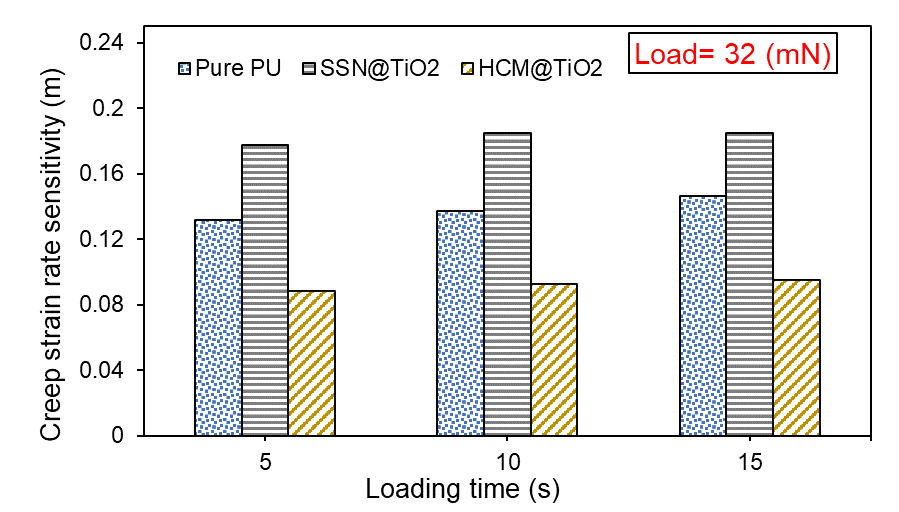 |
| Figure S16. Effect of loading time on creep strain rate sensitivity at indentation load of **a.** 2 mN, **b.** 12.5 mN, and **c.** 32 mN. | |
